# ABMA, a small molecule that inhibits intracellular toxins and pathogens by interfering with late endosomal compartments

**DOI:** 10.1038/s41598-017-15466-7

**Published:** 2017-11-14

**Authors:** Yu Wu, Valérie Pons, Amélie Goudet, Laetitia Panigai, Annette Fischer, Jo-Ana Herweg, Sabrina Kali, Robert A. Davey, Jérôme Laporte, Céline Bouclier, Rahima Yousfi, Céline Aubenque, Goulven Merer, Emilie Gobbo, Roman Lopez, Cynthia Gillet, Sandrine Cojean, Michel R. Popoff, Pascal Clayette, Roger Le Grand, Claire Boulogne, Noël Tordo, Emmanuel Lemichez, Philippe M. Loiseau, Thomas Rudel, Didier Sauvaire, Jean-Christophe Cintrat, Daniel Gillet, Julien Barbier

**Affiliations:** 1 Service d’Ingénierie Moléculaire des Protéines (SIMOPRO), CEA, Université Paris-Saclay, LabEx LERMIT, 91191 Gif-sur-Yvette, France; 2 Service de Chimie Bio-organique et Marquage (SCBM), CEA, Université Paris-Saclay, LabEx LERMIT, 91191 Gif-sur-Yvette, France; 3 Agence Nationale de Sécurité du Médicament et des Produits de santé (ANSM), CTROL/TOMIC, 34740 Vendargues, France; 4 Biocenter, Department of Microbiology, University of Würzburg, 97074 Würzburg, Germany; 5 Antiviral Strategies Unit, Virology Department, Institut Pasteur, 75015 Paris, France; 6 Department of Virology and Immunology, Texas Biomedical Research Institute, San Antonio, 78227 TX USA; 7 ImmunoPharmacology and Biosafety Laboratory, BERTIN Pharma, CEA, 92260 Fontenay-aux-Roses, France; 8 IMAGERIE GIF, Institute for Integrative Biology of the Cell (I2BC), CEA, CNRS, Université Paris-Sud, Université Paris-Saclay, 91190 Gif-sur-Yvette, France; 9 Antiparasitic Chemotherapy, Faculty of Pharmacy, BioCIS, UMR 8076 CNRS, University Paris-Sud, 92296 Chatenay-Malabry, France; 10 Bactéries anaérobies et Toxines, Institut Pasteur, 75015 Paris, France; 11 U1184, Immunology of Viral Infections and Autoimmune Diseases, IMETI, IDMIT, CEA, 92260 Fontenay-aux-Roses, France; 12 INSERM U1065, Equipe Labellisée Ligue Contre le Cancer, Centre Méditerranéen de Médecine Moléculaire (C3M), Université de Nice Sophia-Antipolis, 06204 Nice, France

## Abstract

Intracellular pathogenic microorganisms and toxins exploit host cell mechanisms to enter, exert their deleterious effects as well as hijack host nutrition for their development. A potential approach to treat multiple pathogen infections and that should not induce drug resistance is the use of small molecules that target host components. We identified the compound 1-adamantyl (5-bromo-2-methoxybenzyl) amine (ABMA) from a cell-based high throughput screening for its capacity to protect human cells and mice against ricin toxin without toxicity. This compound efficiently protects cells against various toxins and pathogens including viruses, intracellular bacteria and parasite. ABMA provokes Rab7-positive late endosomal compartment accumulation in mammalian cells without affecting other organelles (early endosomes, lysosomes, the Golgi apparatus, the endoplasmic reticulum or the nucleus). As the mechanism of action of ABMA is restricted to host-endosomal compartments, it reduces cell infection by pathogens that depend on this pathway to invade cells. ABMA may represent a novel class of broad-spectrum compounds with therapeutic potential against diverse severe infectious diseases.

## Introduction

There is a growing need for broad-spectrum drugs to fight existing and emerging infectious diseases (EID) and to be prepared for potential bioterror attacks with toxins or microorganism^1^. Each new EID crisis reveals our level of unpreparedness that is due to the difficulty to predict which pathogen will emerge and to the impossibility to develop new drugs within a few months. A strategy for broad-spectrum drug discovery is the search for molecules targeting host components indispensible for entry and/or multiplication of many different toxins and pathogens into cells^1–6^. Such drugs may have efficacy against unknown pathogens that will emerge in the future. Moreover, molecules active against host cell components should avoid the risk of drug-resistant pathogens^7^.

Plant and bacterial protein toxins acting inside cells, as well as intracellular infectious pathogens such as viruses, intracellular bacteria and parasites have evolved sophisticated strategies to invade host cells that share common features^8–12^. They bind to cell-surface receptors to trigger their internalization. Then, they follow endocytic and intracellular trafficking pathways. Toxins, viruses and sometimes bacteria enter the cell cytosol from specific trafficking compartments^8,9,11^. Other bacteria and intracellular parasites may subvert cell compartments and trafficking components to build a comfortable vacuoles in which nutrients conducive for multiplication are found^10,12^. Thus, small molecules targeting intracellular trafficking pathways (e.g Retro-2^1,13,14^ and EGA^2,15–17^) or host component (e.g amodiaquine^3^, bithionol^4^) exploited by infectious agents exhibit broad anti-infectious actions.

Here, we report the discovery of ABMA, a novel broad-spectrum inhibitor of intracellular toxins and pathogens. ABMA was identified using a cell-based high throughput screen (HTS) against cell intoxication by the plant toxin ricin^14^. ABMA protected mice from nasal instillation of an LD_90_ of ricin. Besides, ABMA protected cells from intoxication by at least four bacterial toxins and from infection by three viruses, two intracellular bacteria and one parasite. In addition, the molecule was not toxic to cells or mice at active concentrations. We further showed that the broad-spectrum anti-pathogenic action of ABMA is associated with the biogenesis of host cells’ late endosomes (LE) without affecting other organelle integrity. Hence, ABMA has the potential to inhibit any toxin or infectious pathogen relying on LE to enter the cytosol or build its intracellular vacuole.

## Results

### Identification by HTS of ABMA, a ricin inhibitor active *in vitro* and *in vivo*

**Figure 1:**
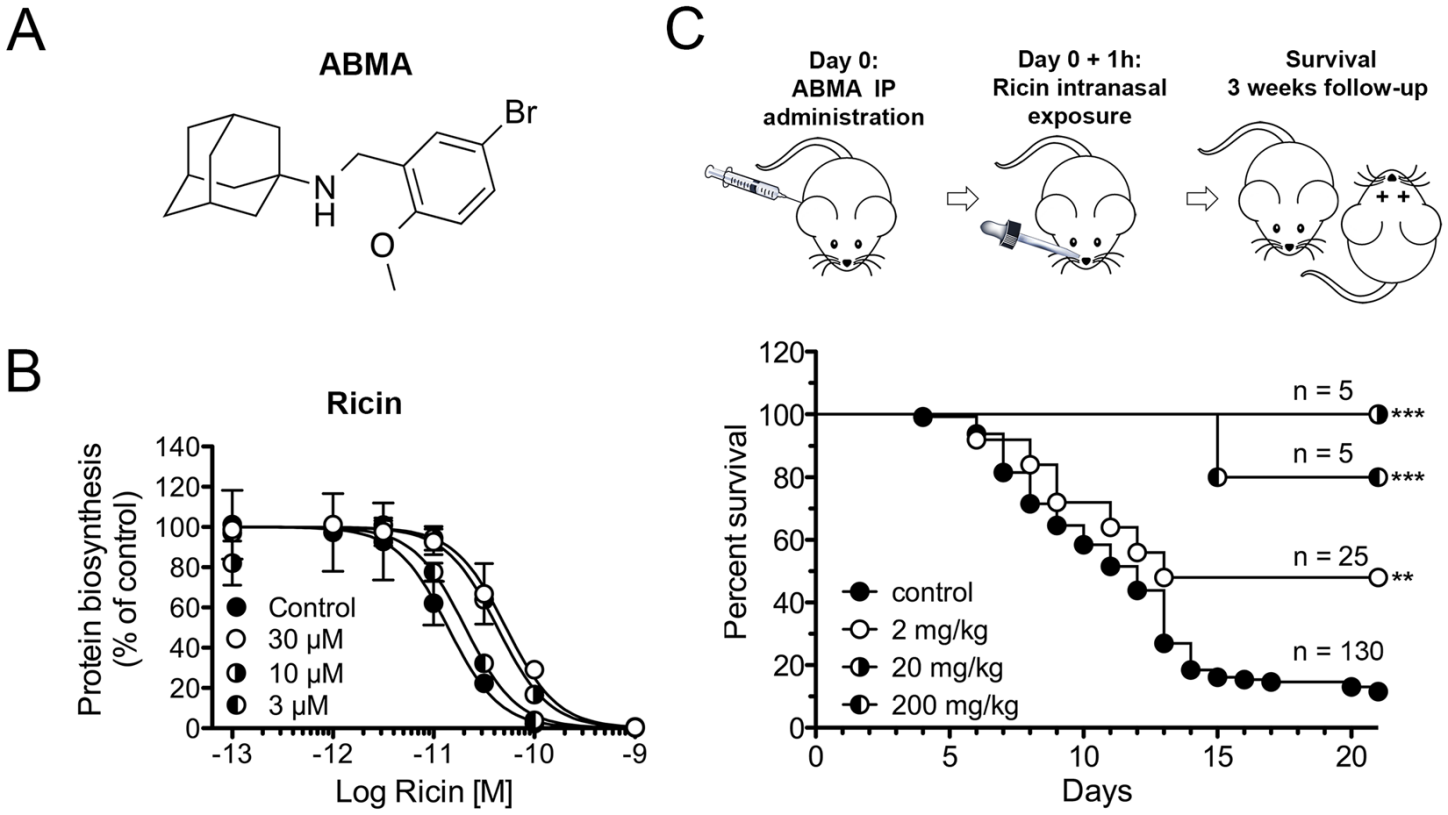
Hit compound ABMA identified as an inhibitor of ricin by HTS. (**A**) Chemical structure of ABMA. (**B**) Intoxication of pulmonary A549 cells by increasing concentrations of ricin in the presence of 3, 10 and 30 µM of ABMA. A549 cells were incubated 4 h in DMEM with ABMA (open and half-filled circles), or solvent only as control (DMSO, black circles) before addition of increasing concentrations of ricin for 20 h. Media was removed and replaced with DMEM containing [^14^C]-leucine at 0.5 µCi/mL for 6 h. Protein synthesis was measured by scintillation counting as the amount of [^14^C]-leucine incorporated in cells. Each data point represents the mean of duplicate ± SD of a representative experiment. (**C**) ABMA protects mice against ricin challenge. The survival of mice treated once with the indicated doses of ABMA and then exposed to an LD_90_ of ricin *via* nasal instillation was monitored. In each experiment, treated animals received a single ip dose of ABMA (2 mg/kg, open circles; 20 mg/kg, circles with right half black; and 200 mg/kg, circles with left half black) 1 h prior to toxin exposure (2 µg/kg by nasal instillation), while control animals (black circles) received vehicle only prior to ricin administration. The curves for treated animals are statistically different from control as measured by the log rank test (p < 0.01 for 2 mg/kg of ABMA; p < 0.001 for 20 mg/kg,; p < 0.001 for 200 mg/kg).

As previously described^14^, we performed a cell-based HTS to identify small chemical compounds active against ricin-mediated cell intoxication. We screened a library of 16,500 small molecules for those endowed with the capacity to prevent the inhibition of protein biosynthesis induced by ricin treatment. Four hits were confirmed; two hits named Retro-1 and Retro-2 were reported as inhibitors of ricin and Shiga-like toxins (Stx) by blocking their retrograde transport inside host cells^14^. ChemBridge^TM^ compound 1-Adamantyl (5-Bromo-2-Methoxybenzyl) Amine (ABMA, Fig. 1A) discussed in this article, was one of the other hits from that screen, which bears a hydrophobic adamantane and a substituted aromatic moiety.

Inhalation is considered as a major risk factor for ricin exposure^18^. Thus, ABMA protective activity was first tested *in vitro* by challenging human pulmonary alveolar basal epithelial A549 cells with increasing concentrations of ricin (Fig. 1B). In five independent experiments, ABMA treatment induced a decrease in ricin cytotoxicity with an EC_50_ of 3.8 µM, and a protection factor (R) at 30 µM ranging from 5 to 10. ABMA retained almost 100% of its biological activity against ricin-induced cytotoxicity up to six days after incubation in culture medium at 37 °C (Fig. S1), indicating a robust stability. As expected, ABMA had no observed inhibitory effect on cell protein synthesis up to at least 90 µM as measured by [^14^C]-leucine incorporation (Fig. S2). AlamarBlue^®^ cell viability assay also confirmed its low toxicity on human cells, with a CC_50_ (50% cytotoxicity concentration) on cultured (HeLa) and primary human cells (Human Umbilical Vein Endothelial Cells, HUVECs) at more than 200 μM (Fig. S3).

Based on these *in vitro* results, we investigated whether ABMA could protect mice against a lethal ricin challenge. ABMA was non-toxic to animals after one intra-peritoneal (ip) administration up to 200 mg/kg. A model of ricin intoxication by nasal instillation^14^ was used to mimic exposure by aerosols, as would occur during an intentional release. Briefly, mice were challenged by an LD_90_ of ricin (2 μg/kg) at day 0 (Fig. 1C, closed circles). The first clinical signs of intoxication appeared within 24 h, all mice displaying bristly and greasy hairs. From day 2, weight loss was observed. At later time points other signs were noticed such as prostration, shaking and respiratory distress, with animals needing to be euthanized starting from day 7 post exposure. A statistically significant protection according to survival curves was observed with a single ip dose of 2 mg/kg of ABMA 1 h prior toxin challenge (p < 0.01 versus control, Fig. 1C, open circles). Forty eight % (n = 25; 3 independent experiments) of ABMA-treated mice survived, while in the control group, survival was 11.5% (n = 130, from 10 independent experiments). Based on this result, additional experiments were performed with escalating doses of ABMA. Administration of a single ip dose of 20 mg/kg and 200 mg/kg of ABMA prior to ricin intoxication gave improved, statistically significant, levels of protection as compared to the control group (p < 0.001, n = 5 for both groups). The 20 mg/kg dose fully protected animals through to day 21 (Fig. 1C). The 200 mg/kg dose resulted in 80% of protection of mice against ricin challenge with a single animal succumbing on day 15. The lower protection seen with the higher dose may be due to solubility issues of ABMA in aqueous solution resulting in uncertain biodistribution at the highest dose.

### ABMA, a broad-spectrum inhibitor active *in vitro* against various bacterial toxins

The mechanism of action of ricin toxin shares common general principles with those of intracellular-acting bacterial protein toxins: binding to a cell-surface receptor, internalization in endocytic compartments, trafficking through intracellular transport pathways, translocation from transport vesicles or compartments into the cytosol and catalytic modification of a cellular target. Thus, investigating the effect of ABMA on bacterial toxins may lead to identify other sensitive toxins and hence, get some insights into ABMA mechanism of action^4,5^. Table S1 summarizes the features of the tested toxins.

**Figure 2:**
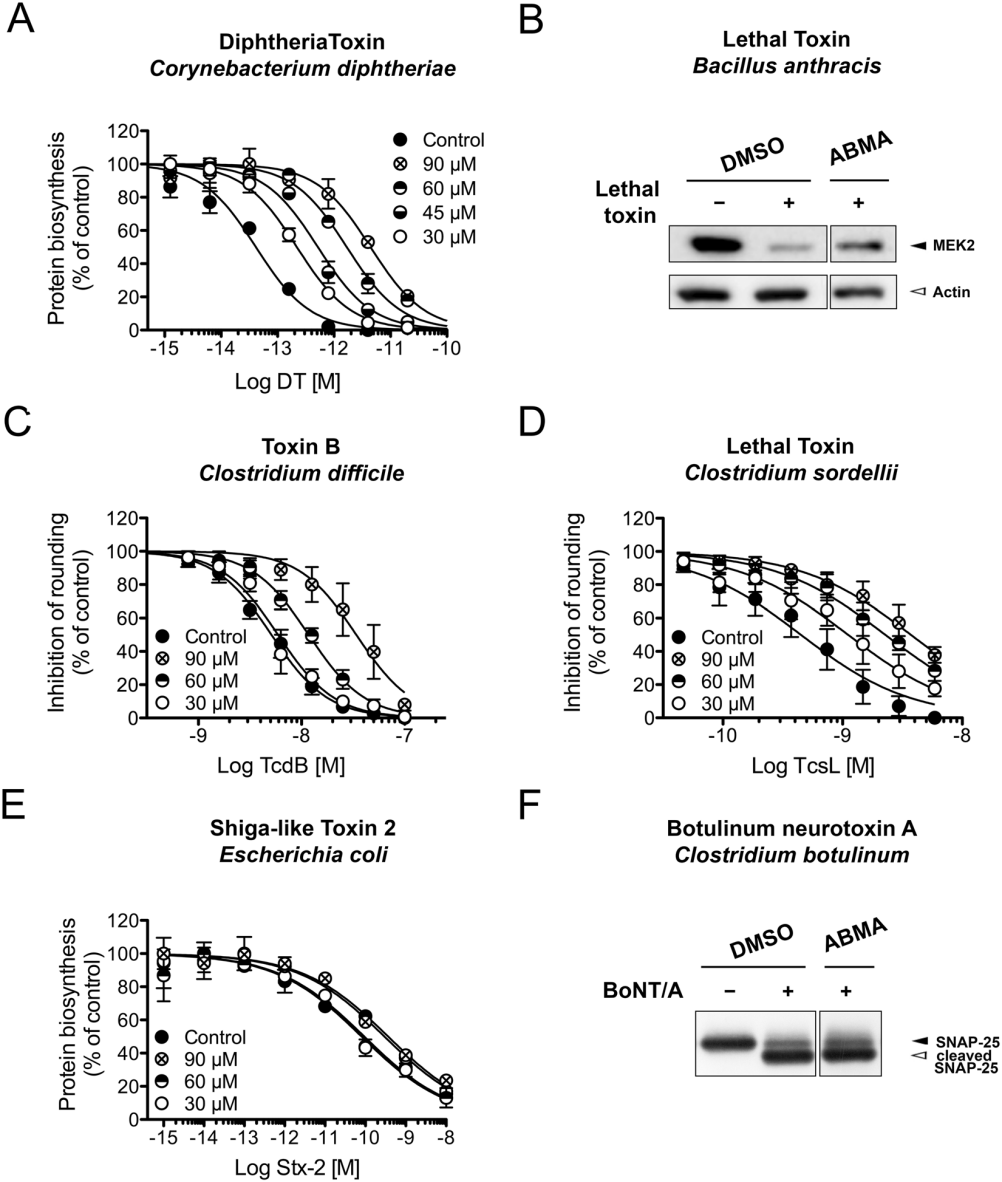
ABMA inhibits cytotoxicity of several bacterial toxins. Cells were incubated with the indicated concentrations of ABMA and then challenged with increasing concentrations of the indicated toxins. (**A**) A549 cells were exposed to DT for 18 h. Culture media was removed and replaced with DMEM containing [^14^C]-leucine at 0.5 µCi/mL for 3 h before protein biosynthesis determination. (**B**) Immunoblots showing the levels of MEK2 in HUVEC cells left untreated (line 1) or treated with Anthrax LT (lines 2–3, LT = PA 3 µg/mL + LF 1 µg/mL) in the absence and presence of 30 µM of ABMA. Immmunoblot of anti-actin show equal protein loading. (**C**,**D**) Vero cells were intoxicated with TcdB for 4 h or TcsL for 18 h and morphological changes of intoxicated cells were imaged and analyzed. (**E**) HeLa cells were exposed to Stx2 for 16 h before protein biosynthesis determination as for DT. (**F**) ABMA or DMSO were added to rat cerebellar granule neurons (CGNs) 1 h prior to BoNT/A exposure (500 pM) in the presence of compounds for 24 h. Immunoblots showing the levels of SNAP-25 and its cleaved form in the absence and presence of ABMA. Immunoblots images from single experiment (**B** and **E**) were spliced to rearrange the order of samples. Full-length blots are presented in Supplementary Figure S8.

Appropriate model cell lines were pretreated with solvent alone (DMSO) or various concentrations of ABMA, then respectively incubated with increasing concentrations of diphtheria toxin from *Corynebacterium diphtheriae* (DT), lethal toxin from *Bacillus anthracis* (LT), toxin B from *Clostridium difficile* (TcdB), lethal toxin from *Clostridium sordellii* (TcsL), Shiga-like toxin 2 from *Escherichia coli* (Stx2) or Botulinum neurotoxin A (BoNT/A) from *Clostridium botulinum* (Table S1 and Fig. 2). The inhibitory effect of DT on protein biosynthesis was measured by the incorporation of [^14^C]-leucine into newly synthesized proteins. We observed higher levels of protein biosynthesis on A549 cells exposed to DT in the presence of ABMA than in its absence (Fig. 2A) with an EC_50_ of 62.5 ± 0.3 µM (n = 3). At 90 µM of ABMA, DT toxicity was reduced more than 100-fold in the assay conditions. We also found that ABMA protected other cell lines (e.g. Vero, PC3, A431 and DLD1) as well as HUVEC primary cells against DT (data not shown). This indicates that the inhibitory effect of ABMA on DT is not cell type-specific. Anthrax LT cleaves the mitogen-activated protein kinase MEK2^19^. Figure 2B shows that MEK2 cleavage by LT in HUVECs was partially inhibited in the presence of ABMA at 30 µM. TcdB and TcsL inactivate small GTPases by their glucosyltransferase activity. This disrupts the actin cytoskeleton and induces cell rounding^20^. ABMA reduced Vero cells rounding 4 and 8 folds, respectively, following a challenge by TcdB for 4 h and by TcsL for 18 h (Fig. 2C,D). The EC_50_s were 73.3 ± 9.1 µM for TcdB and 86.7 ± 6.8 µM for TcsL (n = 3).

Stx2 blocks cell protein biosynthesis by cleaving adenine 4324 of the 28 S ribosomal RNA with the same *N*-adenine glycohydrolase activity as ricin^14^. ABMA at 30 and 60 µM had barely any protective effect on intoxication of HeLa cells by Stx2 and a very weak protection at 90 µM (Fig. 2E). Botulinum neurotoxin A (BoNT/A) cleaves the SNARE protein SNAP25 that is essential for the fusion of neuromediator vesicles to the presynaptic membrane of nerve termini, inducing paralysis of the neuromuscular junction^21^. Figure 2F shows that ABMA at 30 µM was unable to prevent SNAP25 cleavage by BoNT/A in rat cerebellar granule primary cultured neurons, a model for BoNT/A activity.

Taken together, our results show that ABMA had no effect against Stx2 and BoNT/A cell intoxication. Nevertheless, it displays a broad-spectrum antitoxin activity against potent plant and bacterial toxins acting inside cells: ricin, DT, LT, TcdB and TcsL.

Various steps of the toxins’ mechanism of action may be considered as the target of ABMA: receptor binding, internalization, intracellular trafficking, translocation into the cell cytosol and catalytic modification of a cell substrate. The toxins sensitive to ABMA have different catalytic activities: *N*-adenine glycohydrolase for ricin and Stx2, ADP-ribosyltransferase for DT, Zn^2+^ metalloprotease for anthrax LT and BoNT/A and glucosyltransferase for TcdB and TcsL. Thus, it is very unlikely that ABMA inhibits the toxins’ catalytic activities. Each of those toxins uses a different cell-surface component as a receptor for cell binding and internalization (see Table S1). Thus, ABMA probably doesn’t inhibit the binding of the toxins to their receptors. Nevertheless, we investigated whether ABMA could inhibit the binding of DT receptor binding domain, named DTR8^22^, to its receptor pro-HB-EGF (Precursor of heparin binding epidermal growth factor analog) as a model for toxin-receptor interaction^23^. A fluorescent DTR8 was made by chemical coupling with Alexa488 (DTR_A488_). FACS analysis showed that ABMA did not affect binding of Alexa488 labeled-DTR8 (DTR_A488_) with its receptor on Vero cells (Fig. S4), suggests that the inhibition of DT cytotoxicity induced by ABMA is not due to a reduced binding of DT to its receptor.

Since ABMA can inhibit several toxins with different catalytic activities and different receptors, we hypothesized that the inhibitor is not acting directly on the toxin itself but rather on a common host target, necessary for the toxins to reach the host cell’s cytosol and exert their toxicity. Our results demonstrate that ABMA has a pronounced inhibitory effect on DT, LT, TcdB and TcsL, which are all well-characterized acidic endosome-dependent toxins. They require low-pH endosomes (early endosomes (EE) and LE) where they undergo a conformational change, leading to the interaction of their transmembrane and catalytic domains with the compartments’ membrane and translocation of their catalytic domain into the cytosol^8^. In contrast, ABMA had barely any protective effect on the intoxication of HeLa cells by Stx2. Stx2 follows exclusively the retrograde route from the EE to the endoplasmic reticulum (ER) via the Golgi apparatus after internalization into cells^8^. Finally, ABMA is not able to inhibit BoNT/A, which relies on peculiar synaptic vesicle recycling and endocytosis processes to enter into neurons^24,25^. Thus, we hypothesized that ABMA is targeting host’s endosomal trafficking pathway between EE and the lysosomes.

### ABMA inhibits cell infection by viruses that enter the host cytosol from acidified endosomes

**Figure 3:**
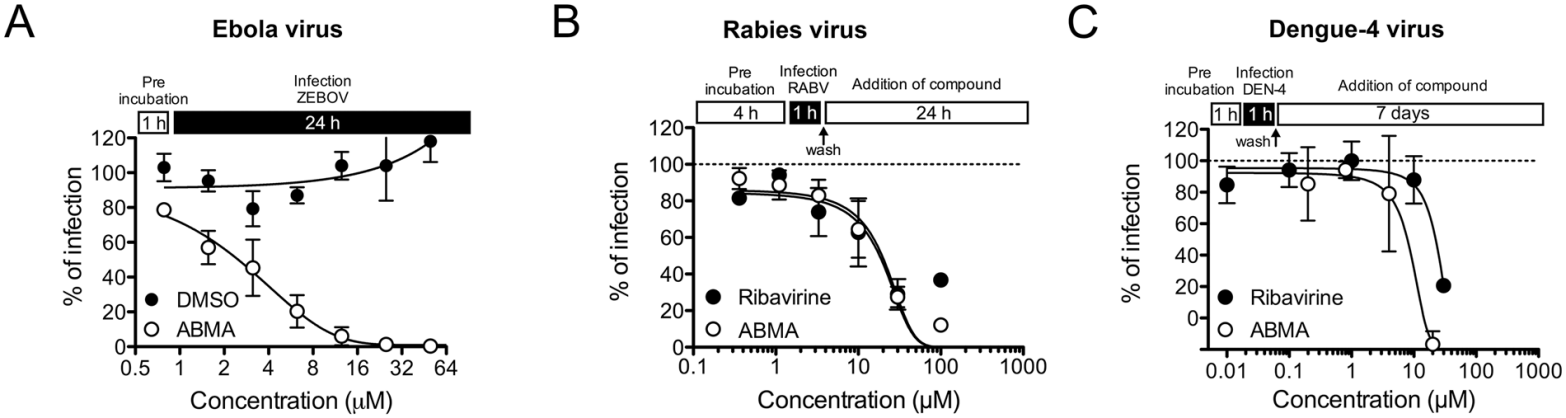
ABMA inhibits EBOV, RABV and DENV4. (**A**) HeLa cells were pre-incubated with increasing concentrations of ABMA solubilized in DMSO, or DMSO only, for 1 h and then challenged with EBOV-eGFP in the presence of the drug for 24 h. Cells were fixed, stained with DAPI, and numbers of nuclei and eGFP-positive (infected) cells were counted using the CellProfiler software. The relative infection efficiencies were calculated by dividing the number of infected cells by the number of nuclei. The percentages of infected cells in DMSO- and ABMA-treated samples were reported relative to the infection efficiency in non-treated cell. Data are representative of three independent experiments. (**B**) BSR cells were pretreated for 4 h with increasing concentrations of ABMA or ribavirin solubilized in DMSO, then challenged with the PV strain of RABV (MOI = 14) for 1 h. Cells were washed to remove the non-fixed virus, then incubated again in the presence of the same concentrations of the compounds for 24 h. Cells were fixed, nuclei were stained with Hoechst and infected cells were detected by immunostaining of the RABV ribonucleocapsid. RABV-positive cells were counted and their number was reported to that of non-treated cell, allowing calculating a percentage of inhibition. The average of three independent experiments and standard deviations are shown. (**C**) Vero cells were treated with ABMA or ribavirin solubilized in DMSO and then challenged with 125 TCID_50_ of a DENV4 serotype virus for 7 days. Viral replication was detected by ELISA using specific serum from DENV4-infected non-human primate.

Knowing that ABMA protects cells from multiple toxins that rely on acidic endosomes to translocate into the cytosol, we investigated whether ABMA was able to inhibit cell infection by viruses that have a pH-dependent mechanism of capsid release from these compartments. We tested Ebola virus (EBOV), Rabies virus (RABV), Dengue-4 virus (DENV4) and Chikungunya virus (CHIKV) (Table S2), which bear a surface glycoprotein that mediates fusion of the virus membrane with that of the endosome after which the capsid is released to the cell cytosol to initiate infection^26^. HeLa cells were incubated 1 h with increasing concentrations of ABMA before infection with a recombinant EBOV Mayinga strain carrying an enhanced green fluorescent protein (eGFP). ABMA treatment inhibited EBOV-eGFP infection with an EC_50_ of 3.3 µM (Fig. 3A). Baby hamster kidney (BSR) cells were incubated 4 h with ABMA before infection with the Pasteur vaccins/PV strain of RABV, ABMA inhibited the infection with an EC_50_ of 19.4 µM (Fig. 3B). Ribavirin, an antiviral drug inhibiting viral RNA synthesis and viral mRNA capping was used as a reference molecule and exhibited a similar EC_50_. Finally, Vero cells were incubated 1 h with ABMA before infection with DENV4, ABMA inhibited infection with an EC_50_ of 8.2 µM, while Ribavirin was about four fold less efficient in protecting cells from the infection (Fig. 3C). ABMA reduced cell infection by the three viruses up to at least 90% at 20 µM for EBOV and DENV4 and at 100 µM for RABV. In contrast, ABMA up to 100 µM did not inhibit infection of HEK293 cells by Chikungunya virus (CHIKV) (Fig. S5, see discussion section). In summary, we observed that ABMA inhibited three endosomal acidification–dependent viruses.

### ABMA inhibits cell infection by *Simkania negevensis* and *Chlamydia trachomatis*

*Simkaniaceae* and *Chlamydiaceae* from the order Chlamydiales are obligate intracellular Gram-negative pathogenic bacteria. They use host-cell materials to form a distinct, degradation-resistant but replication-permissive membranous compartment, the vacuole or inclusion. Despite differences, their intracellular life-styles share several common features. Proteomic characterization of the *Simkania negevensis* (*Sn*) containing vacuole (SnCV) has shown that it contains proteins from several main host transport pathways including the endosomal pathway^13^. *Chlamydia trachomatis* (*Ctr*) recruits multiple Rab proteins from the endosomes to the inclusion membrane and avoids travelling to the phago-lysosome as a final destination^27^. Besides the Golgi apparatus, multi-vesicular bodies (MVBs), also known as LE, are another essential source of cholesterol and sphingomyelin for the development of *Ctr* inclusions^28,29^. Thus, we tested whether ABMA could inhibit the infection of cells by *Sn* and *Ctr*.

**Figure 4:**
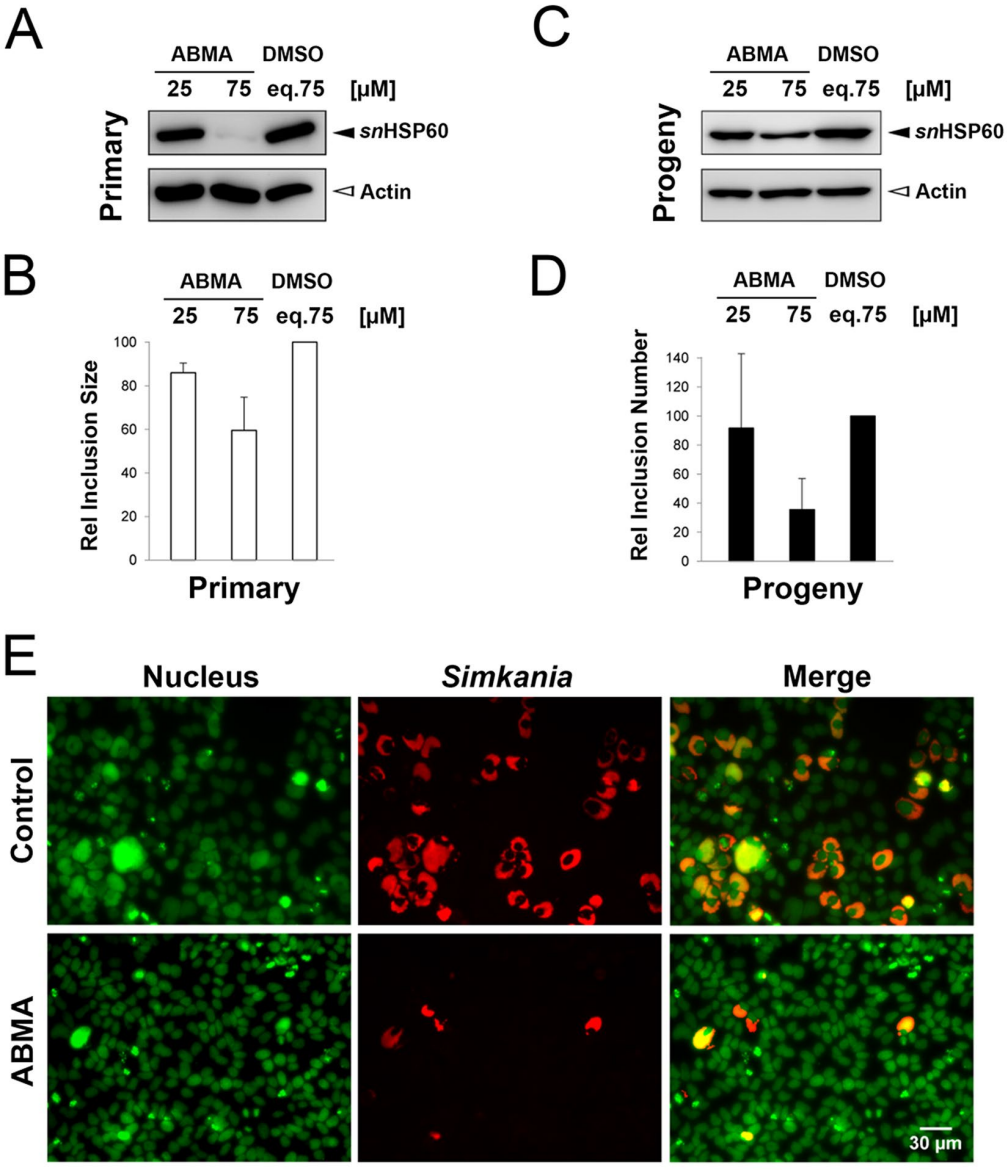
Effects of ABMA on SnCV during *Sn* infection. HeLa 229 cells were infected with *Sn* (MOI = 0.5) for 3 days in the presence of ABMA or DMSO control at the indicated concentrations. Bacterial were released and transferred to infect fresh cells for 3 days in the absence of compounds. (**A**) Effect of ABMA on the *Sn* bacterial load of infected cells measured by *sn*HSP60 immunoblot. Actin was used as loading control. (**B**) Effect of ABMA on the inclusion sizes of *Sn* during primary infection. Relative *Sn* incusion sizes were determined via *sn*HSP60 immunostaining and quantitative analysis using ImageJ. (**C**) Effect of ABMA on the *Sn* bacterial load of progeny infected HeLa cells measured by *sn*HSP60 immunoblot. Actin was used as loading control. (**D**) Effect of ABMA on the number of *Sn* inclusions during progeny infection. (**E**) Immunofluorescence images of cells infected by *Sn* progeny from cells treated with 75 µM ABMA after 3 days of incubation. Nuclei were stained for DAPI (green) and SnCVs were stained for HSP60 (red). Images are representative of 3 independent experiments. Full-length blots (**A** and **C**) are presented in Supplementary Figure S9.

**Figure 5:**
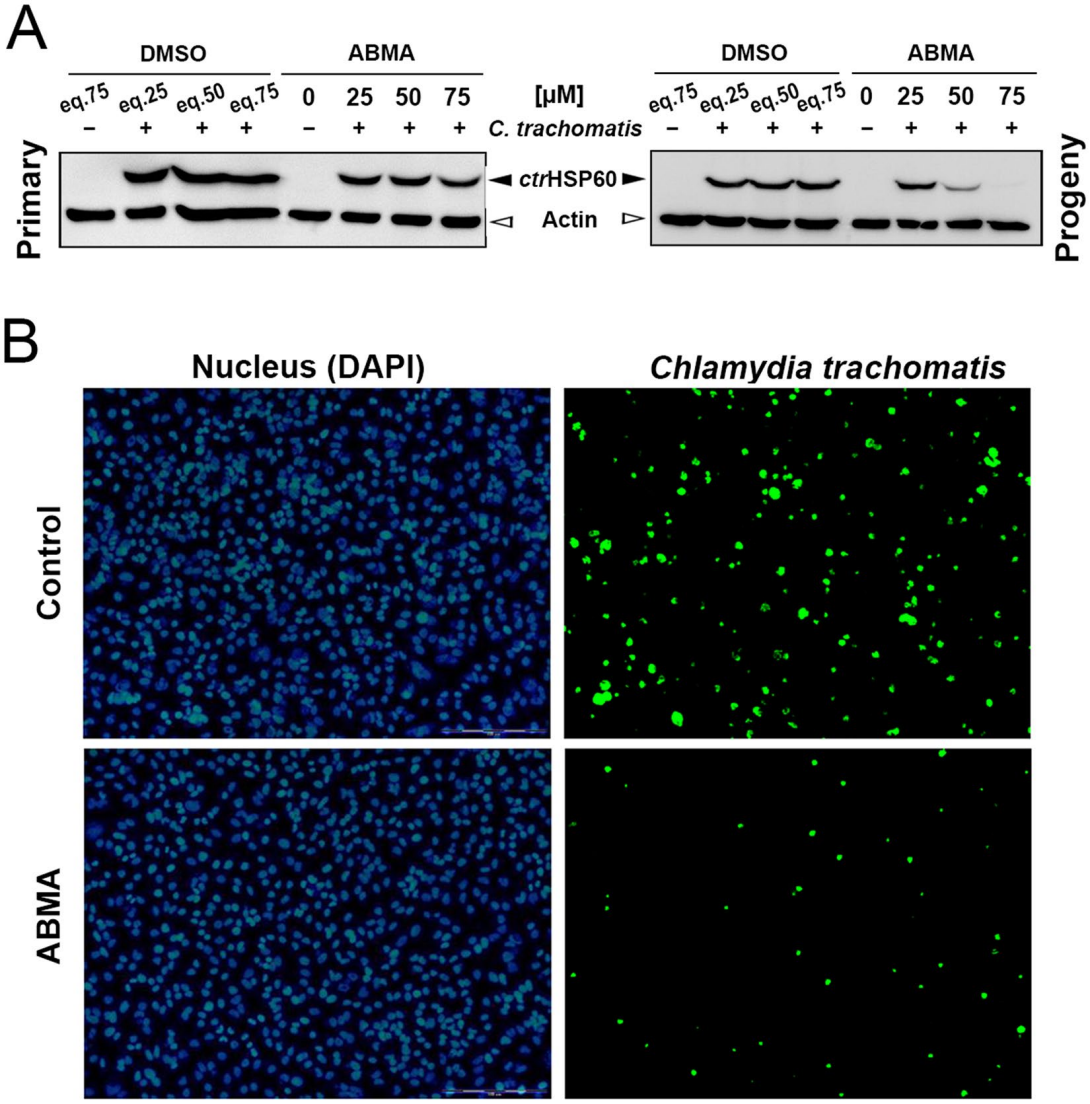
Effects of ABMA on *Ctr* primary and progeny infections. HeLa 229 cells were pretreated with ABMA or DMSO control at the indicated concentrations for 1 hour prior to infection with *Ctr* (MOI = 1). Cells were lysed 48 h post infection and lysates were used to infect fresh cells. ABMA was present during primary infection. (**A**) Immunoblotting analysis of lysed HeLa 229 cells after *Ctr* primary and progeny infections following ABMA treatment during the primary infection. Bacterial load was detected with antibodies against *ctr*HSP60 protein and actin was used as a loading control. (**B**) Immunofluorescence analysis of infectivity with 75 µM ABMA treatment during primary infection. 24 h post progeny infection; cells were fixed and stained for DAPI (blue). *Ctr* inclusions were detected by their GFP-expression signal (green). Immunoblots image (**A**) were spliced to rearrange the order of samples. Full-length blots are presented in Supplementary Figure S10.

Figure 4A shows that 75 µM of ABMA sharply reduced the amount of *Sn* in infected cells as revealed by immunoblotting of *Sn* heat-shock protein 60 (*sn*HSP60). In parallel, inclusion sizes were smaller as revealed by immunofluorescence (Fig. 4B). The *Sn* progeny harvested from ABMA-treated cells reduced the amount of *Sn* (Fig. 4C) and the number of inclusions upon infection of fresh, untreated cells (Fig. 4D,E). ABMA at 75 µM slightly reduced the cellular load of GFP-expressing *Ctr* strain in the primary infection as seen by *Ctr* HSP60 (*ctr*HSP60) immunoblotting (Fig. 5A). However, ABMA treatment dramatically reduced chlamydial progeny infectivity indicated by the reduced inclusion number and bacterial load (Fig. 5A,B). Together, our results show that ABMA inhibits the capacity of *Sn* and *Ctr* to develop properly during cell infection and leads to a progeny with reduced infectivity.

### ABMA inhibits the development of *Leishmania infantum* in macrophages

Monocytes and macrophages are important target cells in the pathophysiology of *Leishmania* parasite infections^30^. The parasite is internalized and develops into an amastigote form within a parasitophorous vacuole that incorporates endo-lysosomal pathway components^30,31^. Thus, we investigated whether ABMA could inhibit the infection of RAW 264.7 macrophages by *Leishmania infantum* amastigotes. Amphotericin B and miltefosine, which are approved drugs for the clinical management of *Leishmaniasis*, were used as reference drugs. Table 1 shows that all three drugs inhibited *L. infantum* intramacrophage amastigotes development with various EC_50s_. The EC_50_ for ABMA was around 7 µM. Interestingly, the two reference drugs were capable of inhibiting axenic amastigotes with an efficacy similar to that found on intramacrophage amastigotes, whereas ABMA had no direct effect on the axenic parasite, up to 100 µM. In addition, the ABMA cytoxicity was lower than those of the reference drugs. These results strongly suggest that ABMA blocks *L. infantum* intracellular development by an action on host cell while the other drugs are directly toxic to the parasite.

**Table 1:** Antileishmanial activity and cytotoxicity of ABMA and reference drugs.

**Compound**	**Axenic amastigotes EC**_**50**_ **(µM) ± SD**	**Intramacrophagic amastigotes EC**_**50**_ **(µM) ± SD**	**Cytotoxicity on RAW 264.7 macrophages CC**_**50**_ **(µM) ± SD**	**Selectivity EC**_**50**_ **(Axenic amastigotes)/EC**_**50**_ **(Intra amastigotes)**
**ABMA**	>100	7.1 ± 1.7	25.3 ± 2.4	>14
**Miltefosine**	1.2 ± 0.5	0.85 ± 0.20	12.5 ± 1.3	1.4
**Amphotericin B**	0.031 ± 0.002	0.047 ± 0.005	4.5 ± 0.4	<1

The results expressed correspond to the mean of three independent experiments (±SD).

### ABMA induces accumulation of late endocytic compartments

**Figure 6:**
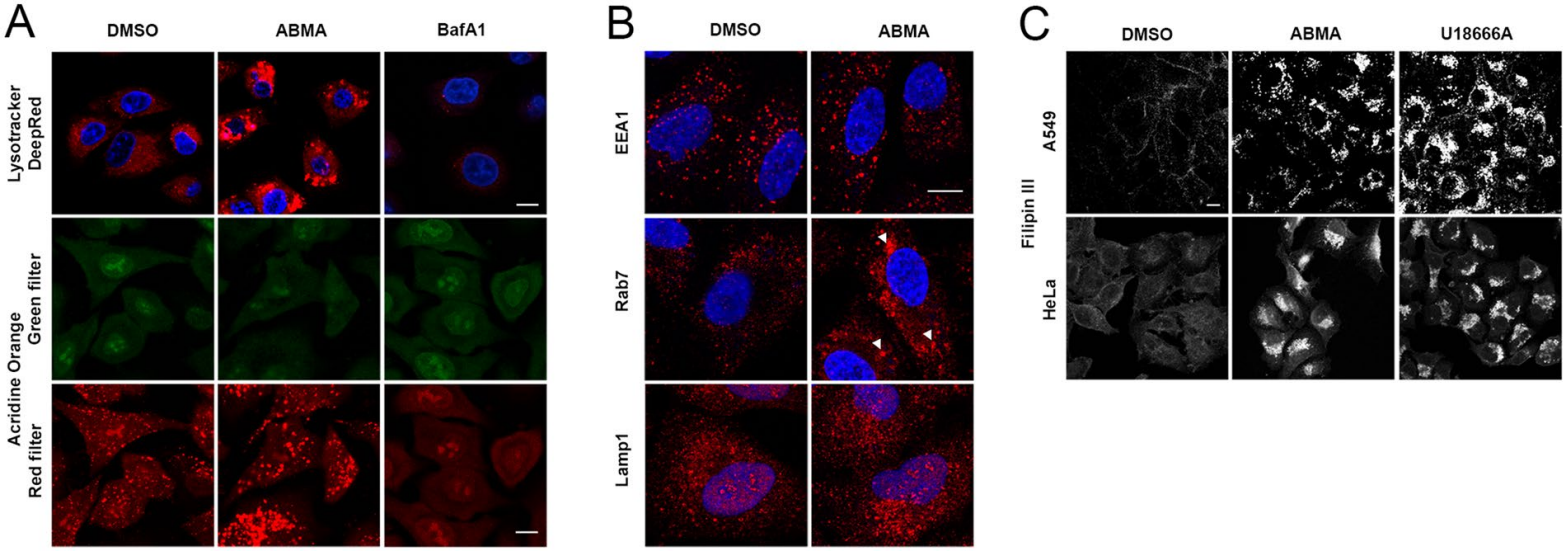
ABMA induces the accumulation of late endocytic compartments and affects cholesterol transport. (**A**) LysoTracker® Deep Red (50 nM, 30 min), acridine orange (10 µg/mL, 10 min) staining of A549 cells pretreated with DMSO or ABMA at 60 µM for 2 h. BafA1 at 100 nM was used as a control. (**B**) DMSO or ABMA 24 h-treated A549 cells were fixed, permeabilized by 0.1% Saponin and stained with antibodies against EEA1, Rab7 or Lamp1. Arrows indicate larger Rab7-positive vesicles. (**C**) A549 and HeLa cells were treated respectively with ABMA (30 µM), U18666A (10 µg/mL) or DMSO for 18 h, then fixed and stained with the cholesterol-avid fluorophore Filipin III. Nuclei were stained with Hoechst 33342 (blue).

We have determined that ABMA can inhibit the intoxication or infection of cells by a variety of toxins, viruses and intracellular microorganisms. The toxins and the viruses rely on endosome acidification to enter the cytosol. The bacteria and parasite build a vacuole that incorporates endosome membranes and proteins to acquire their nutrients and proliferate. This may suggest that ABMA targets and modifies acidic endosomes and their homeostasis. We observed by confocal microscopy that live A549 cells treated with ABMA and stained by LysoTracker® Deep Red exhibited more intensely labeled and enlarged fluorescent puncta than cells treated with DMSO only (Fig. 6A, middle and left panels respectively). In contrast, bafilomycin A1 (Baf A1), a highly specific v-ATPase inhibitor that prevents endosome acidification, decreased fluorescence staining of cells (Fig. 6A, right panel). We obtained similar results with ABMA and Baf A1 on HeLa and RAW 264.7 cells (data not shown). To confirm that ABMA is affecting acidic endosomes, we used acridine orange, another cell-permeant dye for acidic organelles (Fig. 6A, central and lower panels). Similarly, ABMA induced larger and brighter red fluorescent vesicles in A549 cells, in contrast to the effect of Baf A1, which strongly decreased red fluorescence in cytoplasmic vesicles. Altogether, ABMA had an effect on acidic compartments different from that of Baf A1. Baf A1 is known to inhibit DT toxicity by inhibiting endosome acidification. It might be expected that combined effect of both molecules might annihilate the impact of each other: increased endosome acidification for ABMA versus decreased acidification for BafA1. Surprisingly, ABMA combined with Baf A1 had an inhibitory effect on DT cytotoxicity twenty fold higher than for each molecule alone (Fig. S6). This strongly suggests that ABMA and Baf A1 have different mechanisms of action and distinct targets. Moreover, this indicates that the anti-toxin and anti-pathogenic effect of ABMA is not linked to the increase in endosome acidification; otherwise it would be counter balanced by Baf A1.

To further characterize the effect of ABMA on intracellular acidic compartments, immunostaining of protein markers of the EE to lysosomes pathway were performed on A549 cells. Immunostaining of EEA1 (EE marker, Fig. 6B upper panel) and Lamp1 (lysosome marker, Fig. 6B lower panel) were unchanged in ABMA-treated cells and vehicle alone, while Rab7 (LE marker, important GTPase in the late endocytic pathway) staining was visibly enhanced in ABMA-treated cells compared to vehicle alone (Fig. 6B, central panel). Importantly, we did not observe morphological changes on other important cellular organelles such as the Golgi apparatus or ER (stained for Trans-Golgi Network 46 (TGN 46) and Protein disulfide isomerase (PDI), respectively; Fig. S7) as well as endocytosis-related membrane protein (clathrin, adaptin-α, epsin I; data not shown). Altogether, the data show that ABMA targets late endosomal compartments, without affecting the morphology of other organelles: EE, Golgi apparatus, ER and lysosomes.

Besides EE, LE is considered as important and complex sorting stations for proteins and lipids in the endocytic pathway. Cholesteryl esters in LE/Lysosomes are hydrolyzed by lysosomal acid lipase to free cholesterol, before egress from the endo-lysosomal system, allowing for its distribution to other cellular compartments^32^. We investigated if LE modified by ABMA is consequently accompanied by alterations of cholesterol transport, which may potentially interfere with nutrition of intracellular pathogens^12^. Filipin III, a fluorescent probe with high affinity for cholesterol was applied to cells treated respectively with DMSO, ABMA and U18666A, an intracellular cholesterol transport inhibitor. Both ABMA and U18666A induced an accumulation of cholesterol inside A549 and HeLa cells as observed by fluorescent microscopy (Fig. 6C). Together, these data show that ABMA affects LE and induces cholesterol accumulation, likely within late endosomal compartments.

**Figure 7:**
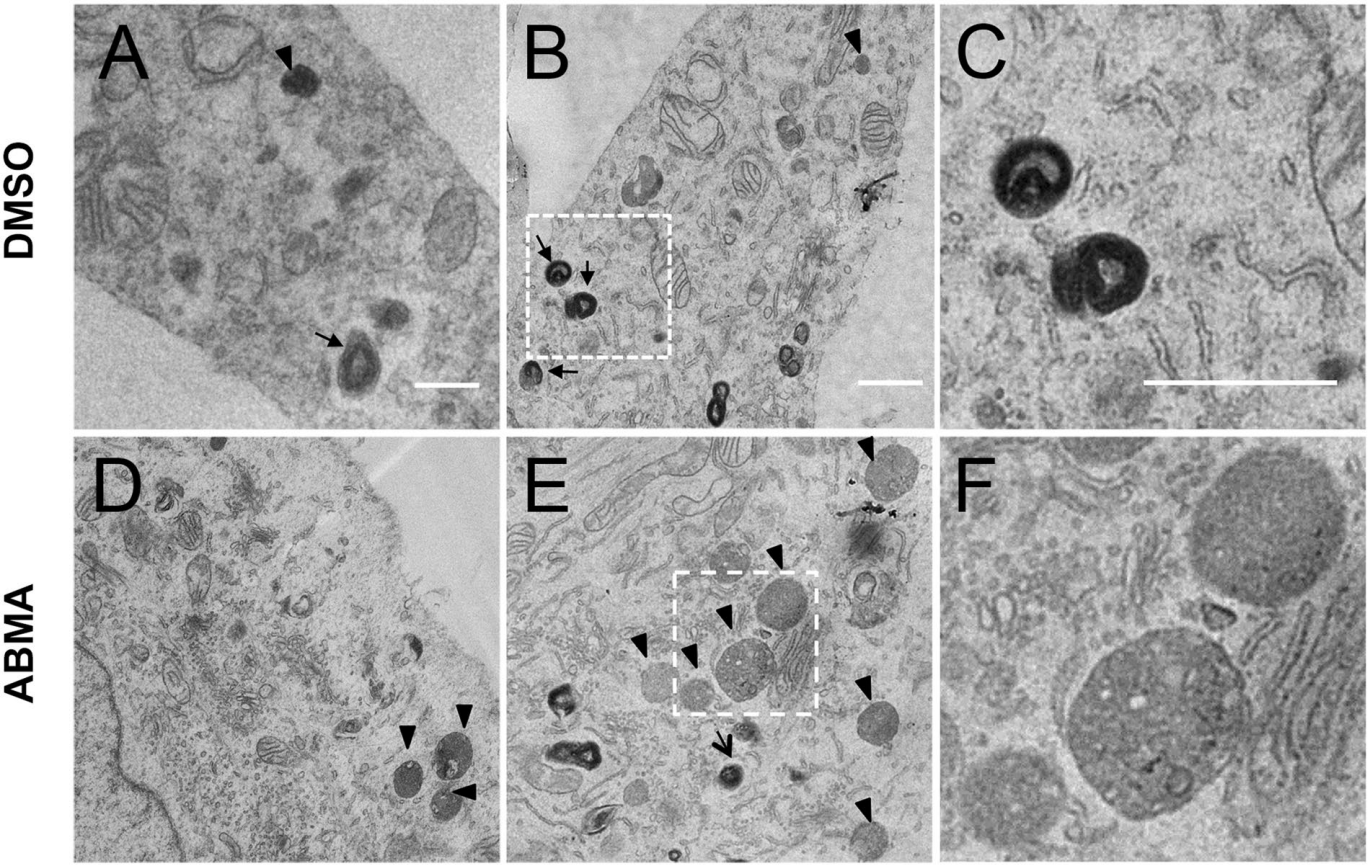
ABMA induces MVBs/LE accumulation. DMSO (**A** and **B**) or ABMA (**D** and **E**) 24 h-treated A549 cells were processed for electron microscopy and representative electron micrographs of sections are shown. Endocytic compartments are marked as follows: MVBs/LE (arrowheads), lysosomes (arrows). Enlarged figures from B and E show representative lysosomes (**C**) and MVBs/LE (**F**).

The enhanced LysoTracker acidification dye and Rab7 staining led us to further investigate how ABMA affects the number and morphology of LE. We used transmission electron microscopy to examine the ultrastructure morphology of organelles in both DMSO- and ABMA-treated A549 cells. We observed that ABMA induced the accumulation of compartments with a >200 nm size, containing a variable number of smaller intraluminal vesicles (ILVs) but lacking a multilamellar morphology (Fig. 7D–F). These structures are characteristic of multivesicular bodies/LE (MVBs/LE)^33^. Other organelles appearing as electron-dense multi-lamellar membrane compartments characteristic of lysosomes were visualized in both DMSO- and ABMA-treated cells without observed difference in amount and morphology (Fig. 7). Altogether, the results show that ABMA induces the accumulation of MVBs/LE.

## Discussion

Here we report that the small molecule ABMA, originated from a cell-based HTS assay to identify ricin inhibitors^14^, protects cells from a wide range of bacterial toxins, viruses and intracellular micro-organisms including bacteria and parasite. The efficacy of ABMA is variable according to the pathogens and toxins investigated. EC_50_ values varied from 60 to 90 µM in the bacterial toxin assays and down to ~7 µM against intra-macrophagic *L*. *infantum* amastigotes or ~ 3 µM against Ebola virus and ricin toxin. However, another key parameter must be taken into account to evaluate efficacy: the level of reduction of intoxication or infection for a given pathogen. For instance, DT toxicity was reduced more than two logs by ABMA; cell infection was decreased below 10% for RABV and down to 0 for EBOV and DENV4. The capacity of *Ctr* progeny to infect new cells was practically abolished by 75 µM of ABMA. All these agents and the corresponding cell assays to measure the compound efficacy are very different, which make comparison difficult. Finally, the ricin *in vivo* assay demonstrates that an inhibitor with a limited efficacy *in vitro* (5 to 10-fold reduction of ricin toxicity) can be enough to protect mice against a lethal toxin challenge. These findings suggest that ABMA may impair Ebola infection in mice, considering its lowest EC_50_ on Ebola among all tested pathogens and toxins *in vitro*.

Several broad-spectrum anti-pathogen compounds have recently been identified from cell-based HTS. They all have direct actions on host cells instead of on the pathogen itself, at µM level^1–5^. Amodiaquine, a clinically approved drug to treat malaria, protects cells against multiple toxins (anthrax lethal toxin, DT, TcdB) and viruses (Ebola, SARS coronavirus, Rabies, Chikungunya) by inhibiting host cathepsin B^3^. Bithionol, an anthelmintic approved drug, inhibits host caspases and also reduces the toxicity of anthrax lethal toxin, DT, cholera toxin, *Pseudomonas aeruginosa* exotoxin A, botulinum neurotoxin, ricin, and Zika virus^4^. EGA, an active molecule against anthrax lethal toxin also blocks *in vitro* trafficking of various toxins (DT, exotoxin A, cytolethal distending toxin, Botulinum Neurotoxins, *Clostridium* toxins) and viruses (influenza virus and lymphocytic choriomeningitis virus) to acidified endosomes^2,15,17^. Retro-2 was proved to have a broad-spectrum action on toxins (ricin, Stx, cholera toxin), viruses (Ebola, Marburg, vaccinia virus, enterovirus 71, adeno-associated virus, polyoma and papillomaviruses), intracellular bacteria (*Sn*, *Ctr*), and parasite (*Leishmania*) by interfering with the intracellular trafficking machinery at the EE-trans Golgi interface^1,34,35^. It is worth noting that ABMA and Retro-2 have demonstrated efficacy *in vivo*. Even if the list of pathogens tested do not overlap in these publications preventing insightful comparisons, distinct mode of action of inhibitors on host cells, and particularly on trafficking pathway, may explain the variety of anti-pathogen spectra observed. Thus, in some instances combinations of these inhibitors may increase, additionally or synergistically, the protection efficacy.

Altogether, our results show that the HTS hit ABMA has a remarkable potential for the development of broad-spectrum drugs against toxins, viruses, and intracellular bacteria and parasites. ABMA being a hit from HTS, there is reasonable hope to obtain other, more powerful, broad-spectrum inhibitors by medicinal chemistry optimization. Activity may be improved both in terms of decreasing the concentration giving maximum efficacy and increasing the level of pathogen inhibition.

ABMA carries a hydrophobic adamantane substituent. Adamantane derivatives have been developed since the 1960s as antiviral drugs against influenza virus infection^36^. However, amantadine, memantine and 1-(1-adamantyl) ethylamine were inactive against DT intoxication and against EBOV infection (data not shown). This indicates that the adamantane group is not sufficient to explain ABMA activity. ABMA and the antiviral adamantine derivatives must display different mechanisms of action to achieve antiviral effects.

The specific cellular target of compound ABMA has not yet been identified. Electron microscopy revealed the intracellular accumulation of MVBs/LE. The increase of LysoTracker, acridine orange and Rab7 staining, as well as the accumulation of cholesterol also demonstrate ABMA acts on the endosome pathway, by inducing the accumulation of acidic late endosomal compartments. Importantly, the integrity of other organelles was unaffected: EE, lysosomes, Golgi, ER and the nucleus.

The nature of the viruses inhibited by ABMA also suggests that LE is targeted by ABMA. EBOV needs the Niemann-Pick disease, type C1 protein (NPC1), a cholesterol transporter and LE/lysosomal protein, to enter cell cytosol^37^. RABV and DENV cell entry are less well characterized. However, it was described that DENV3 must enter Rab7-regulated LE to productively infect Vero cells^38^. In contrast, CHIKV is not sensitive to ABMA. Ninety-five % of CHIKV are internalized through clathrin-coated vesicles, the main internalization pathway for this virus, and then the virus exclusively fuse with EE membranes^39,40^.

LE have long been considered as important sorting stations in the endocytic pathway^41^; they determine whether particular proteins or lipids are targeted to lysosomes for degradation, or alternatively, recycled to the ER or the Golgi apparatus^42^. In addition, MVBs/LE may fuse with the plasma membrane to release exosomes^43^. The endosomal pathway is essential for several toxins and viruses to translocate into the cytosol^41^. Importantly, LE could also supply nutrition (i.e. cholesterol, membrane proteins) for the development of bacteria and parasites inside host cells^30,44,45^. Moreover, similarly to what we observe on human cells with ABMA (Figs 6 and 7), knock-down of Rab7 in HeLa cells results in enlarged MVBs/LE with increased ILVs, which consequently blocks EGFR exiting from MVBs/LE^46^. Thus, the pharmacologic interference of ABMA with the cellular function of Rab7, a key regulator of late endocytic trafficking, might explain its broad-spectrum anti-pathogen activity. Designing drugs that target LE may constitute an advantageous strategy to obtain broad-spectrum drugs against many intracellular pathogens.

U18666A, an inhibitor of cholesterol transport, induces cholesterol accumulation in LE and thereby inhibits toxins (anthrax LT), bacteria (*Chlamydiae*) and viruses (Vesicular Stomatitis Virus, DENV and Hepatitis C Virus)^44,45,47^. ABMA also induces cholesterol accumulation (this work). Both ABMA and U18666A inhibit DT cytotoxicity with similar protection factors (data not shown). Besides, the loss of NPC1 disrupts LE/lysosomes morphology and inhibits EBOV entry^48^. Thus, the mechanism of action of ABMA on host LE may produce multiple consequences to inhibit various pathogens.

In summary, we identified a broad-spectrum chemical inhibitor that hindered several toxins and pathogens by interfering with host specific late endosomal compartments. It protected mice from a lethal ricin challenge without showing toxicity to the animals. Our work highlights that a chemical harboring an action on a cellular component of the host has the potential of a broad-spectrum drug against various pathogens.

## Methods

### Chemicals and materials

1-adamantyl (5-bromo-2-methoxybenzyl) amine (ABMA) was purchased from Chembridge (ID: 5570320, San Diego, CA, USA). Ricin for screening, animal experiments and for *in vitro* validations was supplied by Bruno Beaumelle^14^. Toxin B (TcdB) was produced from *Clostridium* difficile VPI10463, lethal toxin (TcsL) was produced from *Clostridium sordellii* IP82^49^ and anthrax toxin were produced and purified as described previously^50^.

The following products were purchased from the indicated commercial sources: [^14^C]-leucine (Perkin-Elmer); Stx-2 (List Lab, USA); DMSO (D4540), diphtheria toxin DT (D0564), bafilomycin A1 (B1793), Hoechst 33342 (14533), filipin III (F4767), Acridine Orange (A6014), gelatin (G7765) were purchased from Sigma. The following commercial antibodies were used in this study: rabbit anti-Rab7 (ab137029) were from Abcam; Rabbit anti-Rab7 (D95F2) (#9367), rabbit anti-EEA1 (#3288) were from Cell Signaling, Rabbit anti-Lamp1 (L1418) and mouse anti-beta-actin (A2228) from Sigma-Aldrich; rabbit anti-MEK2 (N-20, sc-524), mouse anti-chlamydial trachomatis HSP60 protein (clone A57-B9, sc-57840) from Santa Cruz Technologies; mouse anti-SNAP-25 (SMI-81, 836304) from Biolegend; mouse anti-RNP (ribonucleoprotein) conjugated with FITC from Fujirebio; Alexa 488-donkey anti-rabbit (A10042), Alexa 546-donkey anti-mouse (A10036) and LysoTracker Deep Red (L12492) were from ThermoFisher Scientific.

### HTS

For a full description of the HTS procedure see our previous work^14^.

### *In vivo* experiments

Animal studies were done at French Health Products Safety Agency (ANSM) animal care facility and in compliance with ANSM committee policies according to European regulations. Pathogen-free six week-old female BALB/c mice were purchased from Charles River Laboratories (L’Arbresle, France). Mice were housed under a 12-hour light-dark cycle and fed a standard diet *ad libidum*. Standardized groups of mice were injected intraperitoneally with 500 µL of sterile saline solution (0.9% NaCl) supplemented with 1–10% DMSO alone (control) or with various doses of ABMA one hour prior to toxin administration. Mice were anesthetized by intraperitoneal injection (100 µL) of ketamine (1.15 mg final)-rompun (xylazine, 0.28 mg final) solution and exposed to 50 µL of ricin (2 µg/kg) by intranasal instillation corresponding to one DL_90_ at day 0. Each drug-treated group contained five to ten mice, and normal controls contained ten mice. Survival was recorded daily. Data for mice in each test group were compared to those for untreated ricin-challenged mice by the log rank test (Prism, Graphpad Inc., San Diego, Calif.) and p-values ≤ 0.05 were considered statistically significant.

### Ethics Statement

Animal experiments are carried out in accordance with decrees (Decree No. 2013–118 of 1 February 2013 on the protection of animals used for scientific purposes), national orders (Articles L214-3, R214-87 to R214-10 of the Code rural) and European directives (86/609 EEC of 24 November 1986 and Directive 2010/63/EU of the European Parliament and of the Council of 22 September 2010 on the protection of animals used in For scientific purposes). The ethics committee “Comité d'éthique régional Languedoc Roussillon” CEEA-LR reviewed and approved the study and its protocols under the permit to experiment CEEA-LR-1035.

### Evaluation of broad-spectrum anti-toxin and anti-intracellular microorganism activity

The procedures of all experiments are described in supplementary information, see Supplemental Material and Methods.

### Immunocytochemistry and live staining

Cells were grown on glass coverslips one day before. Following treatments cells were rinsed with PBS, fixed with 4% paraformaldehyde-PBS for 20 min at room temperature. After three times washes with PBS, cells were permeabilized in 0.1% Saponin or 0.2% Triton for 5 min. Subsequently, cells were blocked and stained with antibodies. Finally, the slides were mounted on glass slides and imaged with an inverted microscope (Ti-U, Nikon) or confocal microscope (SP8X, Leica). For live staining, fluorophore labeled-cells in the presence of compound or DMSO were washed and visualized under confocal microscope immediately.

### Electron microscopy

Confluent cultures of A549 cells (ATCC) treated with 30 µM ABMA or DMSO for 22 h were fixed by 2.5% glutaraldehyde in 0.1 M cacodylate buffer (pH 7.4) for 2 h. Cells were scraped, collected and rinsed with 0.1 M cacodylate buffer for 3 times, then the pellets were post fixed with 1% osmium and 1.5% potassium ferrocyanide for 1 h, processed for dehydration in graded ethanol series and infiltrated in Epon resin mixed with propylene oxide. Ultrathin sections (80 nm) were stained with Oolong Tea extracts (OTE) for 30 min, followed by lead citrate staining for 6 min, and examined by JEOL1400 transmission electron microscope operating at 80 kV.

### Data availability

All data generated or analyzed during this study are included in this published article (and its Supplementary Information files).

## Electronic supplementary material

Supplementary information
