# Supplementary material for: ABMA, a small molecule that inhibits intracellular toxins and pathogens by interfering with late endosomal compartments
Source: Sci Rep. 2017 Nov 14;7:15567. doi: 10.1038/s41598-017-15466-7 (PMC5686106; doi:10.1038/s41598-017-15466-7)
Supplement: Supplementary file 1 — Supplementary information [file 41598_2017_15466_MOESM1_ESM.pdf]

ABMA, a small molecule that inhibits intracellular toxins and pathogens by interfering with late endosomal compartments

Yu Wu[1], Valérie Pons[2], Amélie Goudet[1], Laetitia Panigai[3], Annette Fischer[4], Jo-Ana Herweg[4], Sabrina Kali[5], Robert A. Davey[6], Jérôme Laporte[3], Céline Bouclier[1], Rahima Yousfi[7], Céline Aubenque[7], Goulven Merer[2], Emilie Gobbo[1], Roman Lopez[2], Cynthia Gillet[8], Sandrine Cojean[9], Michel R. Popoff[10], Pascal Clayette[7], Roger Le Grand[11], Claire Boulogne[8], Noël Tordo[5], Emmanuel Lemichez[12], Philippe M. Loiseau[9], Thomas Rudel[4], Didier Sauvaire[3], Jean-Christophe Cintrat[2], Daniel Gillet[1]*, Julien Barbier[1]*

[1] Service d'Ingénierie Moléculaire des Protéines (SIMOPRO), CEA, Université Paris-Saclay, LabEx LERMIT, 91191, Gif-sur-Yvette, France
[2] Service de Chimie Bio-organique et Marquage (SCBM), CEA, Université Paris-Saclay, LabEx LERMIT, 91191, Gif-sur-Yvette, France
[3] Agence Nationale de Sécurité du Médicament et des Produits de santé (ANSM), CTROL/TOMIC, 34740, Vendargues, France
[4] Biocenter, Department of Microbiology, University of Würzburg, 97074, Würzburg, Germany
[5] Antiviral Strategies Unit, Virology Department, Institut Pasteur, 75015, Paris, France
[6] Department of Virology and Immunology, Texas Biomedical Research Institute, San Antonio, 78227, TX, USA
[7] ImmunoPharmacology and Biosafety Laboratory, BERTIN Pharma, CEA, 92260, Fontenay-aux-Roses, France
[8] IMAGERIE GIF, Institute for Integrative Biology of the Cell (I2BC), CEA, CNRS, Université Paris-Sud, Université Paris-Saclay, 91190, Gif-sur-Yvette, France
[9] Antiparasitic Chemotherapy, Faculty of Pharmacy, BioCIS, UMR 8076 CNRS, University Paris-Sud, 92296, Chatenay-Malabry, France
[10] Bactéries anaérobies et Toxines, Institut Pasteur, 75015, Paris, France
[11] U1184, Immunology of Viral Infections and Autoimmune Diseases, IMETI, IDMIT, CEA, 92260, Fontenay-aux-Roses, France
[12] INSERM U1065, Equipe Labellisée Ligue Contre le Cancer, Centre Méditerranéen de Médecine Moléculaire (C3M), Université de Nice Sophia-Antipolis, 06204, Nice, France

Corresponding author
Correspondence to Daniel Gillet, daniel.gillet@cea.fr and Julien Barbier julien.barbier@cea.fr

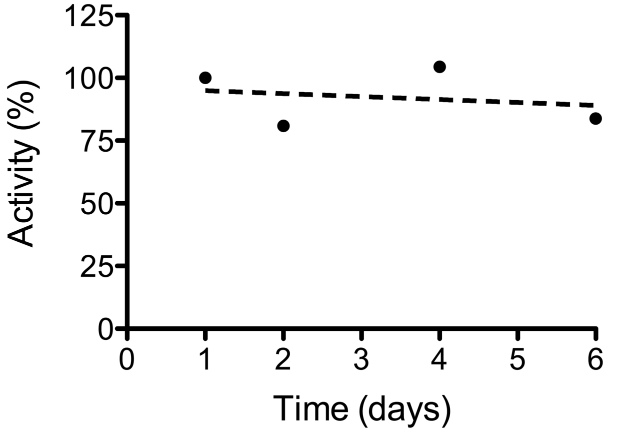

Fig. S1 Stability in culture medium. Aliquots of complete culture medium supplemented with ABMA were prepared, breathable sealed and placed in the incubator, 1, 2, 4 and 6 days before assessing cell protection activity against ricin by protein biosynthesis determination. The biological activity corresponds to the EC_{50} ratio at each time point in comparison to the direct protection assay against ricin-induced cytotoxicity as shown in Fig 1B (100% activity for aliquot prepared 1 day before protein biosynthesis measurement).

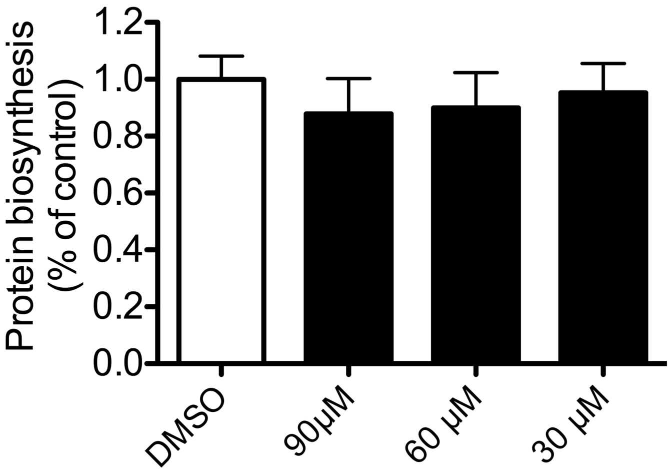

Fig. S2 ABMA does not affect cell protein synthesis.

A549 cells were incubated with ABMA at the indicated concentrations in complete culture medium for 20 h. Protein biosynthesis was determined as in Fig 2A and normalized to cells treated with DMSO alone. Values were from mean ± S.E.M of 4 independent experiments.

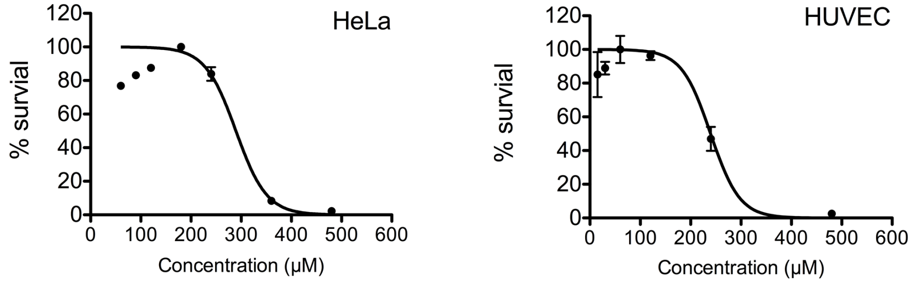

Fig. S3 AlamarBlue® testing of ABMA cytotoxicity on HUVEC and HeLa cells. HUVEC and HeLa cells seeded in 96-well assay plates (black plates, clear bottom with lid) were incubated with increasing concentrations of ABMA in complete culture medium for 20 h. AlamarBlue® reagent was then added as 10% of the culture medium volume and the fluorescence was measured 2 h later with a Cytation 5 cell imaging multi-mode reader (BioTek). Results are from a single experiment.

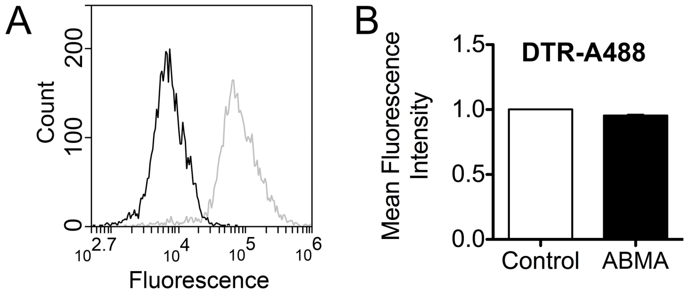

Fig. S4 ABMA has no effect on the binding of DT to its membrane receptor.

DTR8 is a recombinant DT receptor binding domain harboring a mutant in order to improve solubility but without affecting its affinity to receptors[1]. A fluorescent DTR8 was made by chemical coupling with Alexa488 (DTR_{A488}). (A) Flow cytometry of Vero cells incubated at 4°C without (black) or with (gray) DTR_{A488}. (B) Cellular treatment with ABMA at 60 μM or DMSO for 1 h did not affect binding of DTR_{A488} on Vero cells at 4°C ($p>0.05$, $n=3$). Values from mean fluorescence intensity of three independent experiments.

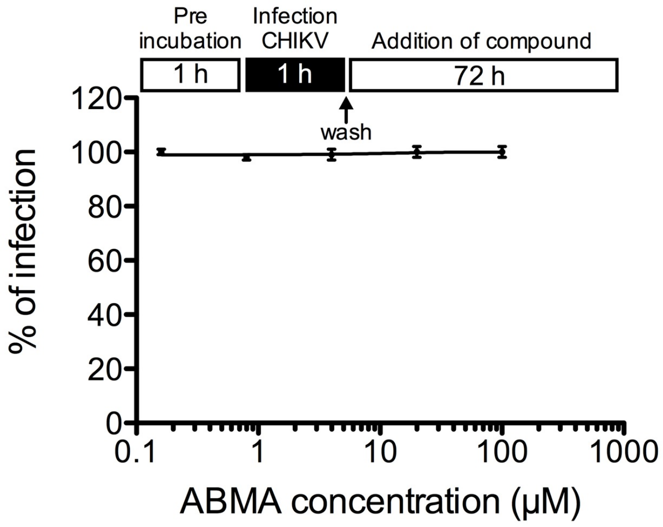

Fig. S5 ABMA does not inhibit infection by CHIKV.

HEK293 cells were pre-treated with ABMA solubilized in DMSO for 1 h and then challenged with CHIKV for 1 h. Cells were washed and then incubated again with same concentrations of ABMA for 72 h. Virus induced-cytopathic effect (CPE) was measured by MTT assay.

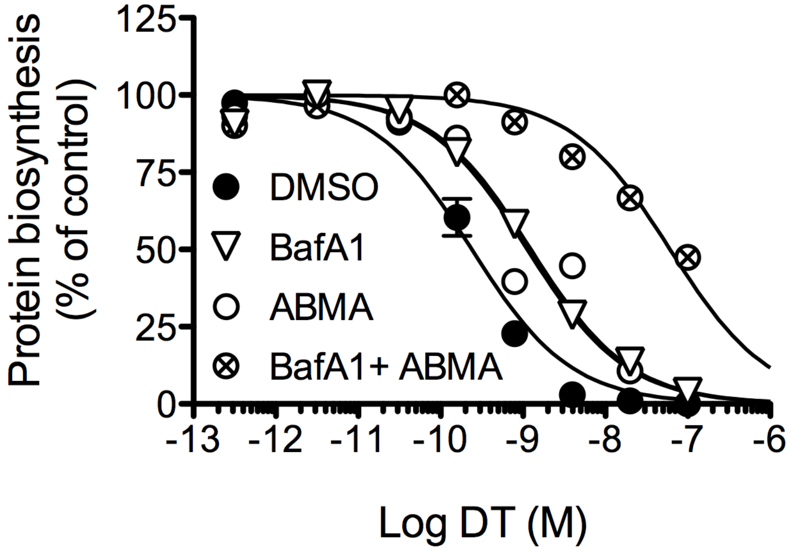

Fig. S6 ABMA together with Baf A1 have apparent synergistic effect to inhibit cytotoxicity of DT. A549 cells were respectively incubated with BafA1, ABMA, both compounds as well as vehicle alone (DMSO) before addition of DT in the continued presence of the compounds. Following steps were performed as described in Figure 2A. The R factor was 4.7 and 5.2 for ABMA and Baf A1, respectively, and reached 63.9 when combined together.

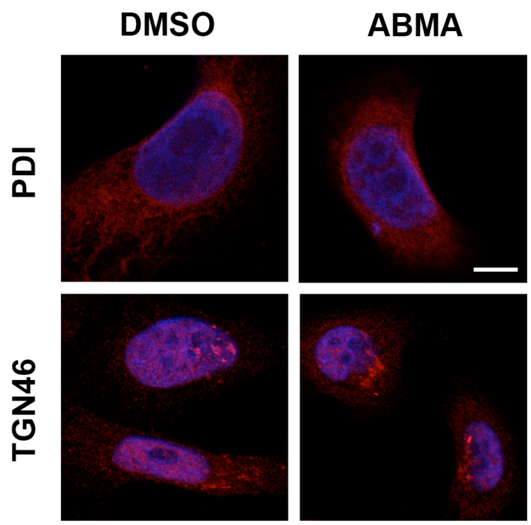

Fig. S7 ABMA does not affect the morphology of the Golgi apparatus or the endoplasmic reticulum.

PC3 cells were incubated with vehicle alone (DMSO) or ABMA (120 µM) for 2 h at 37°C. Cells were fixed, permeabilized and labeled for the indicated compartment markers: PDI (Endoplasmic reticulum), TGN46 (Golgi apparatus). Images were obtained with confocal microscope (SP8X, Leica). Nuclei were stained with Hoechst 33342 (blue). Scale bar, 10 µm.

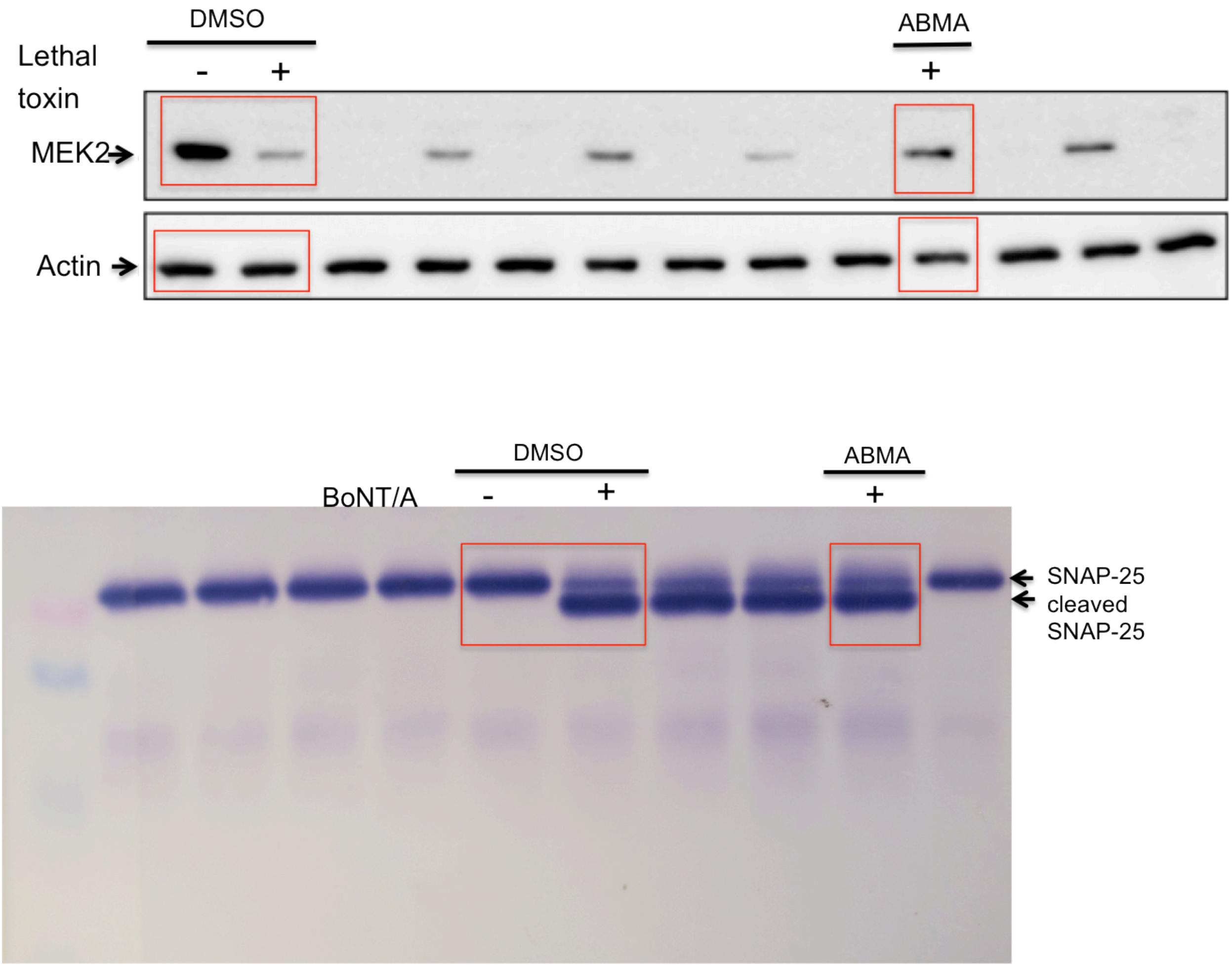

Fig. S8 Full-length blots for Figure 2.

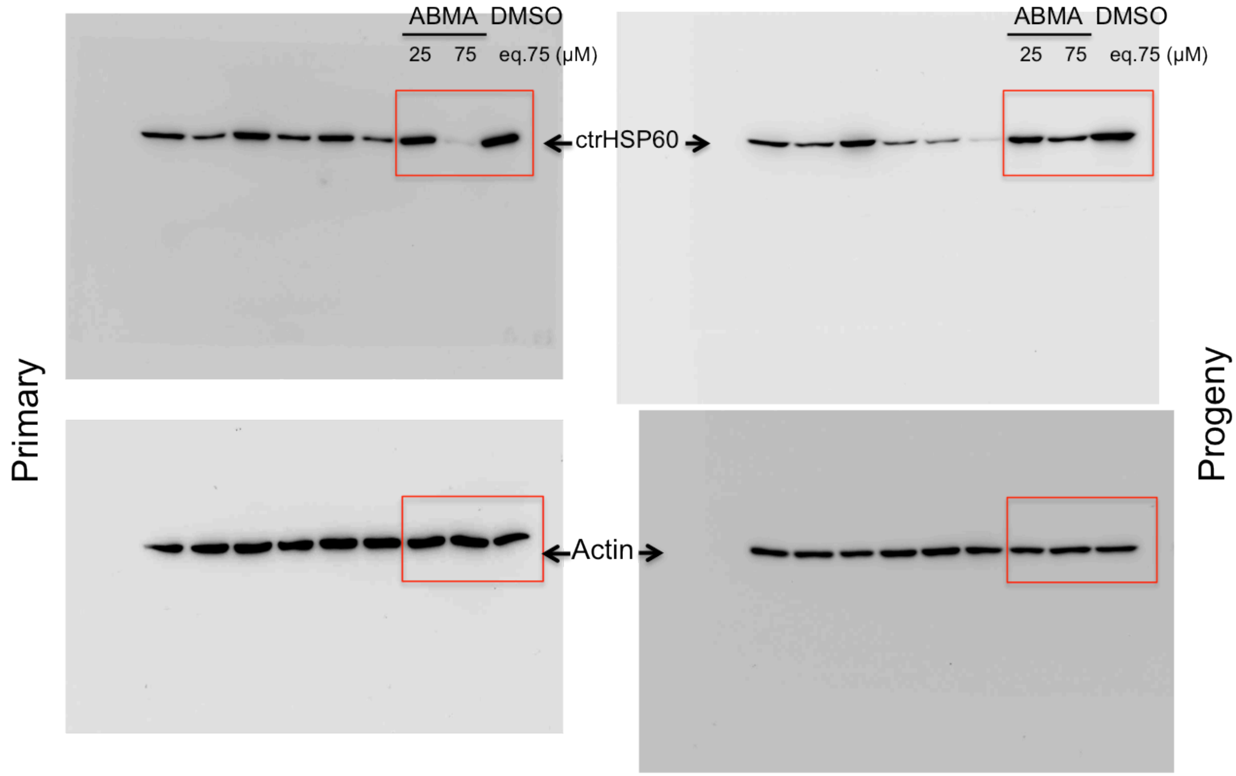

Fig. S9 Full-length blots for Figure 4.

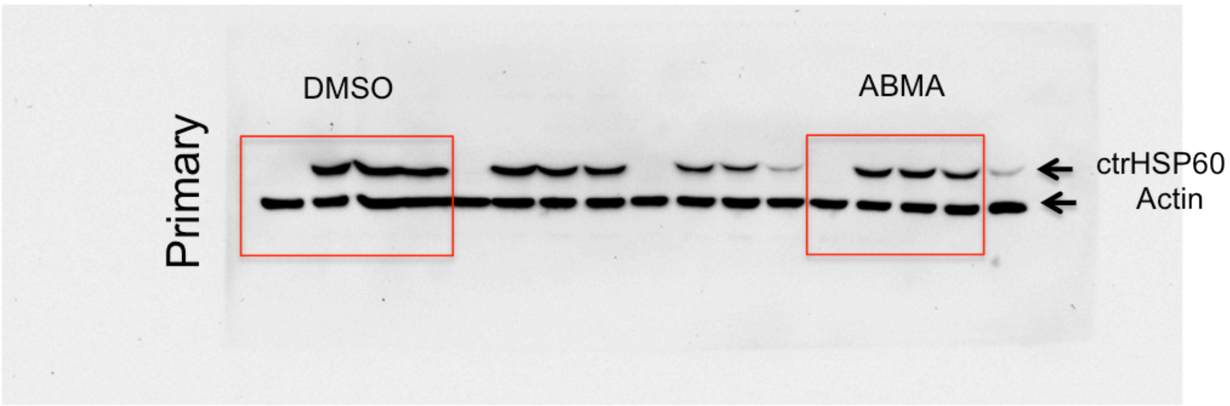

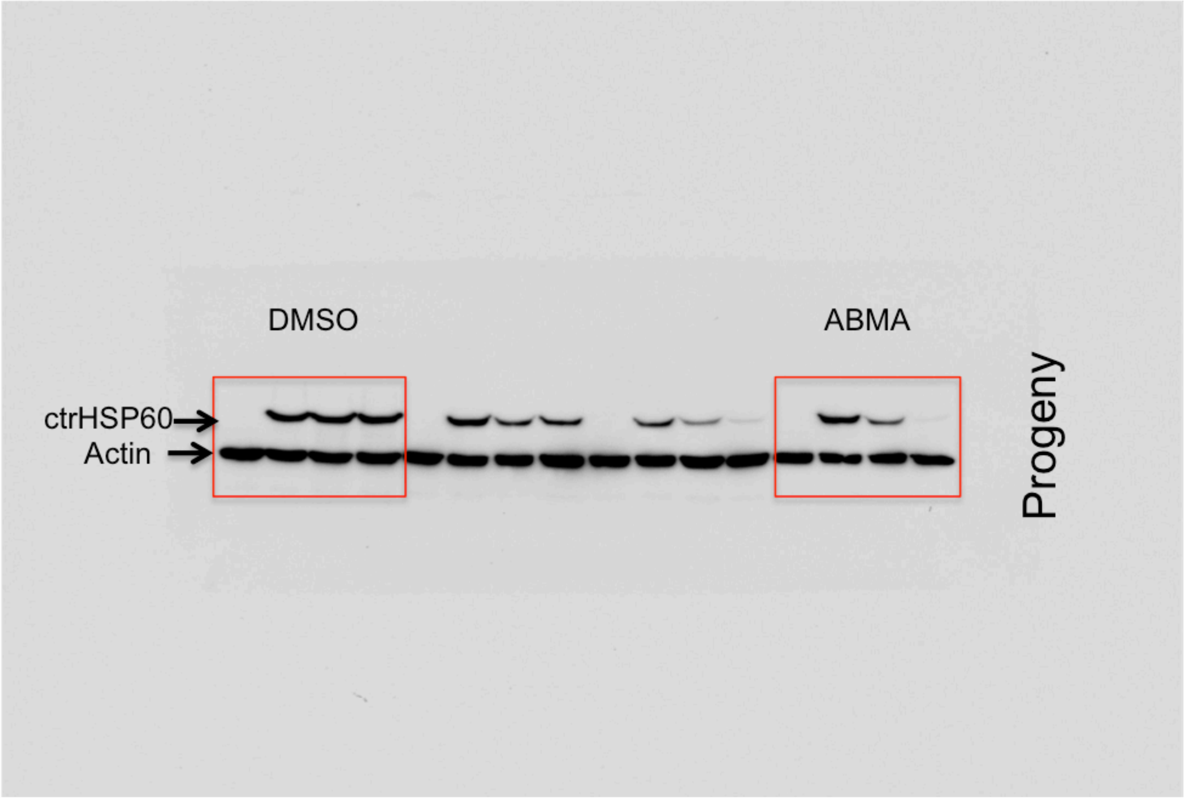

Fig. S10 Full-length blots for Figure 5.

Table S1. Overview of the toxins used in this study

Toxin	Cellular target	Cellular receptor	Clathrin-dependent endocytosis	Endosomal dependent translocation	Effect of ABMA
Ricin	Inhibits protein biosynthesis	Glycolipids Glycoproteins	Yes	Yes/No	+
DT	Inhibits protein biosynthesis	Pro-HB-EGF	Yes	Yes	+
LF	Cleaves MAP kinase kinases	ANTXR1/ TEM8 ANTXR 2/ CMG2 + LRP6	Yes	Yes	+
TcdB	Glycosylates small GTPases	CSPG4 PVRL3 FZDs	Yes	Yes	+
TcsL	Glycosylates small GTPase	?	Yes	Yes	+
Stx1/2	Inhibits protein biosynthesis	Gb3/Gb4	No	No	-
BoNT/A	Cleaves SNAP-25	SV2	SVR[a]	Yes/No	-

Pro-HB-EGF, Precursor of heparin binding epidermal growth factor analog; ANTXR, Anthrax toxin receptor; TEM8, Tumor endothelial marker 8; CMG2, Capillary morphogenesis gene 2 protein; LRP6, Lipoprotein receptor-related protein 6; CSPG4, Chondroitin sulfate proteoglycan 4; PVRL3, poliovirus receptor like 3; FZDs, Wnt receptor frizzled family; Gb3/Gb4, Glycosphingolipid globotriaosylceramide ; SV2, Synaptic vesicle protein 2.

[a] SVR: Synaptic Vesicles Recycling, depending on neuronal cells, which may or not use clathrin-mediated endocytosis.

Table S2. Overview of the viruses used in this study

Virus	Family	Genus	Genome	Envelope	Main cellular receptor	Endosomal - dependent translocation	Effect of ABMA
Ebola Zaire EBOV	*Filoviridae*	Ebolavirus	negative single-stranded RNA	Yes	NPC1 (in endosome) TIM-1	Yes	+
Rabies RABV	*Rhabdoviridae*	Lyssavirus	negative single-stranded RNA	Yes	nAChR NCAM LNGFR	Yes	+
Dengue-4 DENV4	*Flaviviridae*	Flavivirus	positive single-stranded RNA	Yes	DC-SIGN (CD209) Mannose receptor (CD206)	Yes	+
Chikungunya CHIKV	Togaviridae	Alphavirus	positive single-stranded RNA	Yes	PHB, PVEERs, GAGs	Yes（95% within early endosome）	-

NPC1, Niemann-Pick C1; TIM-1, T cell immunoglobulin mucin domain 1; nAChR, Nicotinic acetylcholine receptors; NCAM, neural cell adhesion molecule; LNGFR, low-affinity nerve growth factor receptor; DC-SIGN (CD209), dendritic cell-specific adhesion receptor; PHB, Prohibitin; PVEERs, Phosphatidylserine (PtdSer)-mediated virus entry-enhancing receptors; GAGs, Glycosaminoglycans.

SUPPLEMENTAL MATERIALS AND METHODS

Evaluating efficacy on toxins by protein synthesis assay

Assay. Human HeLa cells (ATCC) or A549 cells were maintained at 37°C under 5% CO2 in DMEM (Dulbecco's modified Eagle's medium, Invitrogen), supplemented with 10% fetal bovine serum (12476-016, Gibco), 100U/mL penicillin, 100 µg/mL streptomycin (15140-122, Gibco), 0.1 mM non-essential amino acids (11140-035, Gibco). HUVECs (PromoCell, Heidelberg, Germany) were maintained at same conditions in human endothelial SFM (11111-044, Gibco), supplemented with 20% fetal bovine serum, 20 ng/mL bFGF (13256-029, Gibco), 10 ng/mL EGF (13247-051, Gibco), 1 µg/mL Heparin (H3149, Sigma) and 100 U/mL penicillin - 100 µg/mL streptomycin (15140-122, Gibco).

The cells were plated in 96-well Cytostar-TTM scintillating microplates (Perkin-Elmer) with scintillator incorporated into the polystyrene plastic one day before. After incubation with various concentration of compounds (or carrier, DMSO) for indicated time at 37 °C, cells were challenged with increasing doses of ricin, diphtheria toxin or Stx2 for at least 16 h in continued presence of compounds. Subsequently, the medium was removed and replaced with DMEM without leucine (Gibco) containing 10% fetal bovine serum, 2 mM L-glutamine, 0.1 mM non-essential amino acids, 100 U/mL penicillin - 100 µg/mL streptomycin supplemented by 0.5 µCi/mL [^{14}C]-leucine. The cells were grown for an additional 3-6 hours at 37 °C in an atmosphere of 5% CO2 and 95% air. Protein biosynthesis was then determined by measuring the incorporation of radiolabeled leucine into cells using a Wallac 1450 MicroBeta liquid scintillation counter (Perkin Elmer).

The mean percentage of protein biosynthesis was determined and normalized from duplicate wells. All values are expressed as means ± SEM. Data were fitted with Prism v5 software

(Graphpad Inc., San Diego, CA) to obtain the 50% inhibitory toxin concentration (IC_{50}) i.e. the concentration of toxin that is required to inhibit 50% protein synthesis of cells. IC50 values and protection factor R (R = IC_{50} drug/IC_{50} DMSO) were determined by the software's nonlinear regression "dose-response EC_{50} shift equation". The goodness of fit for toxin alone (carrier) or with drug was assessed by r^2 and confidence intervals.

Determination of EC_{50} values of ABMA against ricin, DT, Stx2. EC_{50} was used to evaluate ABMA efficacy because it is more precise than R values and associated % protection. This is due to the fact that R values may fluctuate between cell experiments from different 96-wells plates corresponding to compound tested on different days. In contrast EC_{50} value for a single compound is calculated from experimental data obtained on a single 96-well plate. Cell assays were performed with various concentrations of the compound. For each concentration, a percentage of protection was determined from R values calculated with Prism software with Rmax corresponding to the higher value of R of the series:

$$\% \text{ protection} = \frac{R - 1}{Rmax - 1} \times 100$$

Drug concentration was plotted against the corresponding percentage of protection of cells and the 50% efficacy concentration (EC_{50}) was calculated by non-linear regression using the Prism software package.

Cell rounding induced by large clostridial glucosylating toxins on cultured cells

Cytotoxic effects of the TcdB and TcsL toxins on Vero cells (Sigma-Aldrich) were assessed by cell rounding assays. Vero cells seeded in 96-well plates at a density of 10,000 cells per well one day before exposuring to increasing doses of TcdB (4 hours) or TcsL (16 hours) in the continued

presence of compounds at various concentrations. Cell rounding was visualized by phase-contrast microscopy using an inverted microscope (Ti-U, Nikon) with a CFI Achro Ph ADL-10X/0.25 objective. Approximately 100-200 cells, counterstained with Hoechst 33342 dye, were counted and the percentage of rounded cells was determined. EC_{50} was calculated by non-linear regression using the Prism v5 software.

Anthrax toxin MEK cleavage Assay

Cell lysates from HUVECs intoxicated with LT (3 µg/ml protective antigen (PA) and 1µg/ml Lethal factor (LF)) in the absence or presence of ABMA were resolved on 12% SDS-PAGE gels using standard conditions and transferred to Immobilon-P PVDF membranes (Millipore). The antibodies used were as follows: an antibody directed toward the amino-terminal part of MEK2 (MEK2-N20, Santa Cruz), anti-beta-actin (Clone AC74, Sigma). The primary antibodies were revealed using horseradish peroxidase-conjugated goat anti-mouse or anti-rabbit secondary antibodies (DAKO) followed by chemiluminescence using Immobilon Western (Millipore). The chemiluminescent signals were recorded on a FUJIFILM LAS-3000, and the data were quantified using the MultiGauge V3.0 software.

BoNT/A SNAP25 cleavage assay

Primary cultures of rat cerebellar granule neurons (CGNs) were prepared from 6-days-old rats as previously described [2]. Briefly, cerebella were isolated, mechanically disrupted and then trypsinized. Cells were then collected and plated into 24 well plates, pre-coated with poly-L-lysine (50 µg/ml), at a cell density of 5×10^5 cells per well. Cultures were maintained at 37 °C, 5% CO_2, 95% humidity in neurobasal medium supplemented with B27, to which 20 mM KCl,

0.5 mM glutamine 100 U/ml penicillin and 100 U/ml streptomycin were added. To inhibit proliferation of non-neuronal cells, cytosine β-D-arabinofuranoside (5 μM) was added to the medium 24 h after plating. ABMA (30 μM) was added to cell medium 1 h prior to BoNT/A exposure (500 pM, 24h). Cell lysate were collected and SNAP-25 protein was detected by immune blot with antibody (SMI-81, Biolegend).

EBOV experiments

All experiments with replication competent EBOV were performed in the biosafety level 4 (BSL-4) laboratory at the Texas Biomedical Research Institute (San Antonio, TX). The recombinant Zaire EBOV strain Mayinga expressing enhanced GFP (EBOV-eGFP) was kindly provided by Heinz Feldmann (NIH, Hamilton, MT). The viruses were amplified in Vero-E6 cells (CDC, Atlanta, GA) for 7 days in DMEM containing 2% FBS. The culture supernatants containing the viruses were clarified of cell debris, overlaid over a 20% sucrose cushion in PBS, and centrifuged at 4°C at 28,000 rpms for 2 h. The pellets were resuspended in PBS. The titer of EBOV-eGFP stock was determined by incubating serial dilutions of the stock on Vero cells for 24 h. Infected cells were fixed with 10% buffered formalin (Sigma, St. Louis, MO) for 24 h, and then stained with Hoechst 33342 dye (Life Technologies, Carlsbad, CA) to identify cell nuclei. Cells were photographed using a Nikon Ti-Eclipse microscope running high content analysis software (Nikon, Tokyo, Japan). The numbers of cell nuclei and infected cells were counted using CellProfiler software (Broad Institute, Boston, MA) with pipelines developed by the authors (available upon request).

EBOV infection. To test impact of ABMA on virus infection, HeLa cells grown in 96-well plates in MEM medium supplemented with 10% fetal bovine serum and incubated at 37°C, 5%

CO_2. Cells were either left untreated or pretreated in triplicate with the compound or DMSO in 2-fold serial dilutions for 1 h and then challenged with EBOV-eGFP, at a multiplicity of infection (MOI) of 0.1 for 24 h. This virus is replication competent and shows normal pathogenesis in animals but expresses GFP 24 hours after infection has taken place. For this reason, the experiment was halted at 24 hours after virus challenge, which was sufficient time for a single round of virus infection and expression of the GFP infection marker. Subsequently, infected cells were fixed in 10% formalin and stained with DAPI. The infection was then analyzed by photographing each well using an epifluorescent microscope. Total and infected cells were counted by counting DAPI stained cells and green fluorescent cells respectively using CellProfiler software. The infection rate was calculated as the ratio of infected cells to cell nuclei.

$$\text{fraction infection} = \left(\frac{\text{number of green fluorescent cells}}{\text{number of DAPI stained cell nuclei}}\right)$$

$$\text{inhibition (\%)} = 100 \times \left(1 - \frac{\text{fraction infection after drug treatment}}{\text{fraction infection without treatment}}\right)$$

Three parameter dose response curves were fitted to data by non-linear regression and EC50 values determined using Graphpad Prism 5 software. The equation used is below:

$$\mathrm{Y} = \text{lower plateau} + (\text{upper} - \text{lower plateau}) \div \left(1 + 10^{(\mathrm{X} - \log \mathrm{IC50})}\right)$$

***In vitro* RABV infection experiments**

Assay. BSR cells (a clone of BHK cells, gift of Monique Lafon) were incubated in DMEM with 8% FBS and 40 µg/mL of gentamycin for 24 h at 37°C and 5% CO_2 in 96-well plate (35,000 cells/well). After washing with DMEM, cells were pre-incubated for 4 h at 37°C in DMEM with 2% SVF and 40 µg/mL of gentamycin in presence of ABMA or ribavirin. After 4 h of pre-incubation with compounds, cells were infected with the PV strain of RABV (MOI=14, seed lot also grown on BSR cells) for 1 h at 37°C and 5% CO_2. Cells were then washed once with DMEM, then incubated again in DMEM with 2 % FBS and 40 µg/mL of gentamycin in presence of compound at the same concentration. After 24h, the supernatant was removed and kept at -80 °C for titration, cells were washed with PBS $^{Ca2+, Mg2+}$, then 80% acetone was added for fixation for 30 min at 4°C. Cells were then incubated with a mouse monoclonal anti-RNP (ribonucleoprotein) conjugated with FITC diluted in PBS for 1 h to detect the cytoplasmic accumulation of intracellular RABV RNP. Hoechst in PBS (1/2000) was used for nucleus staining.

Image acquisition and analysis. Images were acquired on an automated spinning disk confocal microscope (Opera QEHS, Perkin Elmer Technologies) at the Imagopole (Institut Pasteur imaging platform). Briefly, 15 fields were acquired per well with a 10× air objective (NA 0.4) to reveal the nuclei stained with Hoechst (405 nm/450 nm) and to detect the RABV-RNP revealed by a fluorescein-coupled antibodies (488 nm/540 nm). The image analysis was performed using AcapellaTM (Perkin Elmer Technologies) as follows: (1) the nuclei were segmented in the Hoechst channel to estimate the number of cells imaged in each well (above 10,000 total nuclei – TN - in the non-infected control wells); (2) the segmentation mask was then increased to the limit of each individual cytoplasm, generating a “cell mask” in which the viral RNP (green dots) were segmented using a spot detection module of the software, allowing the reporting of the

number of infected cells – InfC – i.e. containing at least 1 green spot. Finally, we calculated the ratio [InfC/TN*100], which reports the percentage of infection of each well.

DENV4 infection experiments

Vero cells (ATCC) were plated into 96-wells microplates for 30 min pre-treated with 6 concentrations of ABMA and one reference compound ribavirine, and infected with 125 $TCID_{50}$ per well of a DEN-4 serotype virus. Seven days later, cells were lysed using Igepal 2.5% in phosphate buffer (PBS) before quantifying viral replication using specific ELISA conditions (a specific serum from DENV-4-infected non-human primate and peroxidase-conjugated goat anti-monkey IgG diluted in PBS, 5% milk, 0.5% Tween 20). Absorbance was measured using a spectrophotometer at 492 nm.

Previously, this viral strain was amplified and titrated by limit-dilution; the 50% tissue culture infectious dose ($TCID_{50}$) was calculated using Kärber's formula.

CHIKV infection experiments

HEK 293 (ATCC) were plated into 96-wells microplates 24 h before. Cells were pre-treated with different concentrations of ABMA for 1 h and then infected with CHIKV for 1 h. Cells were washed and incubated again with same concentration of ABMA for 72 h. Virus induced-cytopathic effect (CPE) was measured by MTT assay.

***Sn* infectivity assay**

40,000 HeLa229 cells (ATCC) were seeded in 12-well cluster plates, compound-treated and infected as indicated in the respective experiment. Cells were either fixed and stained or

harvested for immunoblotting at indicated time points or bacteria were released via one freeze thaw cycle (-70°C/37°C) followed by mechanical release through pipetting and transfer to fresh HeLa229 cells (1:25-1:50, progeny/infectivity). Cells were centrifuged for 1 h at 35°C and medium exchanged to infection medium. Progeny was fixed at day 3 post infection and processed for staining or harvested for immunoblotting. Infectivity assays were imaged on an automated fluorescence microscope Leica DMIR. Numbers and average sizes of the SnCV as well as host cell numbers were determined via snHsp60 and DAPI staining and images were analysed and quantified using FIJI (Image J) and Excel (Microsoft).

In this assay, bacteria are first grown in cells treated with compound (ABMA at a concentration of 25 and 75 μM) and the infectious particles from this primary infection are applied to fresh cells in the absence of compounds to measure the bacterial load (snHSP60 immunoblotting) and inclusion formation (immunofluorescence microscopy).

Immunoblotting and immunofluorescence staining. Western blotting was performed according to standard procedures and signals were detected with a Chemo Cam Imager (Intas). Quantification was performed using FIJI (ImageJ) and Excel (Microsoft). For immunofluorescence, cells were seeded in 12-well cluster plates with or without coverslips, and were infected with MOIs indicated in the respective experiments in a humidified incubator at 35°C and 5% CO_2. Cells were fixed at indicated time points with 4% PFA-sucrose for 25 min, washed once with PBS. Subsequently cells were permeabilized with 0.2% Triton X-100 in PBS for 45 min and washed 3 times with PBS for 15 min at RT. Cells were blocked with 2% goat serum in PBS for 60 min and were stained with primary antibodies diluted in 2% goat serum in PBS for 24 h at 4°C. After 3x washing with PBS for 15 min, samples were incubated with secondary antibodies for 1 h in blocking solution at RT in the dark. Cells were washed 3 times

with PBS and counterstained with DAPI (1 μg/mL) in PBS for 15 min. Coverslips were mounted with Mowiol 4–88 (Carl Roth) after short wash with H_2O. 12-well cluster plates without coverslips were washed 3 times with PBS for 15 min and visualized at RT. Samples were subsequently viewed on a Leica DMIR or TCS SP5 microscope, respectively. Data were processed using FIJI (Image J). Antibodies against snHsp60 were prepared as previously described [3].

Ctr infectivity assays

HeLa229 were grown in RPMI1640 medium (Glutamax, 10% heat-inactivated FBS, w/o HEPES) (Invitrogen). Cells were pretreated with ABMA in concentrations of 25, 50 and 75 μM for 1 h until Ctr (MOI=1) were added to the cells. ABMA was present during primary infection. To obtain progeny infection, compound-treated cells were lysed with glass beads 48 h post infection (hpi) and lysate was used to infect fresh HeLa229 cells. Progeny infection in absence of ABMA was lysed 24 hpi and analyzed together with primary infection samples by immunoblotting. Chlamydial growth was detected with antibodies against ctrHSP60 (clone A57-B9, Santa Cruz Biotechnology) and β-actin (clone AC-15, Sigma-Aldrich) was used as loading control. Immunofluorescence analysis of Progeny infection was also performed 24 hpi, cells were fixed and stained for DAPI. Ctr inclusions were detected by their GFP-expression signal.

In vitro antileishmanial evaluation

The experiments were performed by using the *Leishmania infantum* strain (MHOM/FR/2008/LEM5700). Antileishmanial activity on *L. infantum* axenic amastigotes was carried out as previously described [4].

Antileishmanial activity on the *L. infantum*/RAW 264.7 macrophage model. As previously described [5], mouse macrophage cells RAW 264.7 (ATCC) were seeded into a 96-well microplate and maintained in DMEM medium (Applied Biosystems, France) supplemented with 10% heat-inactivated fetal bovine serum in a 5% CO2 incubator at 37 ºC for 24 h. Then, the medium was replaced with 100 µL fresh medium containing a suspension of 10^5 *L. infantum* amastigotes and the incubation time was 16 h. Then, extracellular parasites were eliminated from the medium and 100 µL fresh medium containing compound in various concentrations were added for a 48 h-incubation. Parasite growth was determined by using SYBR® Green I (Life technology, Fisher Scientific, France), a dye with marked fluorescence enhancement upon contact with DNA. The cell lysis suspension was diluted 1:1 in lysis buffer (10 mM NaCl, 1 mM Tris HCl pH8, 2.5 mM EDTA pH 8, 0.05% SDS, 0.01 mg/mL proteinase K and 10 × SYBR Green I). Incorporation of SYBR Green I in DNA amplification was measured using the Master epRealplex cycler® (Eppendorf, France) according to the following program to increase the SYBR green incorporation: 90°C for 1 min, decrease in temperature from 90°C to 10°C for 5 min with reading the fluorescence at 10°C for 1 min and a new reading at 10°C for 2 min. The EC_{50} as the concentration inhibiting the parasite growth by 50% was calculated by nonlinear regression using icestimator website 1.2 version: http://www.antimalarial-icestimator.net/MethodIntro.htm. Amphotericin B, miltefosine were used as reference drugs. The concentrations used for drug evaluation were in a range from 0.05 to 100 µM, obtained by serial dilution with a factor of two, except for amphotericin B, for which the range was from 0.005 to 10 µM.

Cytotoxicity evaluation on RAW 264.7 macrophages. RAW 264.7 cells were seeded into a 96-well microtiter plate at a density of 10^5 cells/well in 100 µL of DMEM. After incubation in a 5% CO_2 incubator at 37°C for 24 h, the culture medium was replaced with 100 µL of fresh DMEM

containing the compounds for a new incubation of 48 h. The final compound concentrations used were from 0.05 µM to 100 µM obtained by serial dilution. Triplicates were used for each concentration. The viability of the macrophages was then assessed using the SYBR® Green I (Invitrogen, France) incorporation method and the results were expressed as cytotoxic concentration inhibiting the cell growth by 50% (CC_{50}) in µM.

Flow cytometry

Mutant receptor binding domain of diphtheria toxin (DTR8) was produced in house [1] and fluorescent DTR8 (DTR_{A488}) was obtained using Alexa Fluor labeling kit (A20181, Thermo Fisher). Cell surface toxin receptors (proHB-EGF) were incubated and labeled with DTRA488 (1:50 dilution, on trypsinized Vero cells) in PBS/BSA 1% at 4°C prior to incubate with compound for 1 h. After rinsing with the same buffer, single cell suspension was subjected to FACS analysis. Analysis was performed by flow cytometry (Accuri C6 cytometer, BD Biosciences) and CFlow Plus software (BD Biosciences).

SUPPLEMENTAL REFERENCES

1 Gillet, D., VILLIERS, B., Pichard, S., Maillere, B. & Sanson, A. Hb-egf inhibitor derived from the R domain of diphtheria toxin for the treatment of diseases associated with the activation of the HB-EGF/EGFR pathway (US Patent 9,758,552, 2017).

2 Wong, J. K., Kennedy, P. R. & Belcher, S. M. Simplified serum- and steroid-free culture conditions for high-throughput viability analysis of primary cultures of cerebellar granule neurons. *Journal of neuroscience methods* **110**, 45-55 (2001).

3 Mehlitz, A. *et al.* The chlamydial organism *Simkania negevensis* forms ER vacuole contact sites and inhibits ER-stress. *Cell Microbiol* **16**, 1224-1243, doi:10.1111/cmi.12278 (2014).

4 Audisio, D. *et al.* Synthesis and antikinetoplastid activities of 3-substituted quinolinones derivatives. *European journal of medicinal chemistry* **52**, 44-50, doi:10.1016/j.ejmech.2012.03.003 (2012).

5 Balaraman, K. *et al.* *In vitro* and *in vivo* antileishmanial properties of a 2-n-propylquinoline hydroxypropyl beta-cyclodextrin formulation and pharmacokinetics via

intravenous route. *Biomedicine & pharmacotherapy = Biomedecine & pharmacotherapie* **76**, 127-133, doi:10.1016/j.biopha.2015.10.028 (2015).
